# Enhanced Transport in Organic Semiconductors Mediated Through Interfacial Charge Transfer and Cooperative Strong Coupling

**DOI:** 10.1002/nap2.70197

**Published:** 2026-07-24

**Authors:** Kuljeet Kaur, Nitin Yadav, Shashi Kumari, Jino George

**Affiliations:** ^1^ Molecular Strong Coupling Lab Department of Chemical Sciences Indian Institute of Science Education and Research (IISER) Mohali Mohali Punjab India

**Keywords:** charge transport, electron mobility, light‐matter interaction, polaritonic states, polaritronics

## Abstract

Band structure engineering of organic semiconductors is crucial to achieve the best‐performing optoelectronic devices. Strong light–matter coupling is emerging as an unconventional approach to tailoring the band structure through exciton–photon hybridization. This work demonstrates that cooperative strong coupling enhances transport in a bottom‐gate, bottom‐contact metal‐oxide‐semiconductor field‐effect transistor (MOSFET)‐based Fabry–Pérot cavity. Here, the cavity consists of an electrically active layer made of a perylenediimide dye and an inactive, highly absorbing cooperative layer (Rhodamine‐B). A large enhancement in electron mobility of the active layer is observed when placed in direct contact with ionic Rhodamine‐B. A feeble enhancement is observed in a lossy mirrorless configuration. The cooperative effect still works here, but it is less pronounced due to weak coupling strength. Placing an insulator between the layers prevents charge transfer. Furthermore, replacing the ionic layer with neutral Perylene‐Red dye does not affect the electrical properties even when the layers are in direct contact. Here, the improved electrical response is due to interfacial charge transfer and strong coupling that work in tandem. These findings highlight how cooperative strong coupling can serve as an effective strategy for engineering the band structure of organic semiconductors and thereby improving their performance.

## Graphical abstract

Here, we experimentally demonstrate the effect of cooperative strong coupling to improve the electron mobility of an organic semiconductor. This is achieved by strong coupling between a highly absorbing organic dye and an organic semiconductor in a MOSFET cavity configuration. Our findings suggest the flexibility of applying cooperative strong coupling to improve material properties.

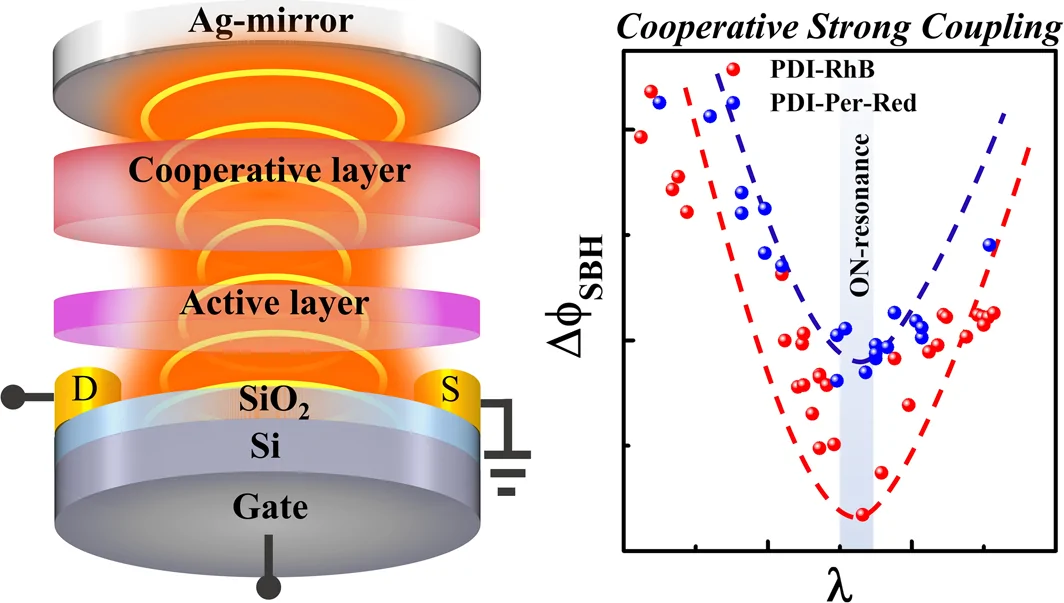

## 1 Introduction

Strong light–matter coupling has emerged as a versatile platform for manipulating the optical, electronic, and chemical properties of materials [1, 2, 3]. This phenomenon is particularly intriguing, as the photon exchange rate between light and matter surpasses their respective dissipation processes, leading to the formation of hybrid light–matter states that possess both excitonic and photonic characteristics. The new hybrid states enable unprecedented control over energy flow and reaction dynamics, among other properties, in ways that are inaccessible in conventional systems [4]. This is due to the collective interaction of many molecules in a cavity that amplifies these effects, leading to several important applications [5]. This includes catalyzing chemical and photochemical reactions [6, 7, 8, 9], enhanced charge [10, 11, 12, 13, 14, 15, 16, 17, 18] and energy transport [19, 20, 21, 22, 23, 24, 25, 26], controlling magneto transport [27, 28], work function modification [29], etc. Strong coupling can be achieved by utilizing various structural designs, such as optical cavities [30, 31], plasmonic structures [32], or photonic crystals [33], etc., and has recently emerged as a powerful tool for engineering material properties beyond conventional limits [34, 35, 36].

Cooperative strong coupling (CSC) extends this concept by simultaneously coupling two different oscillators to a single cavity mode. This phenomenon is advantageous when one oscillator alone is too weak to enter into strong coupling regime. By coupling cooperatively with a stronger oscillator, the properties of the weaker one can be modified through their collective interaction with the cavity field [37]. For example, cooperative vibrational strong coupling involves the collective coupling of reactant and solvent molecules to the same cavity mode when both possess vibrational states at the same frequency [38]. Such cooperative interactions can be harnessed to probe and control reaction kinetics, strengthen intermolecular coupling, and modulate chemical reaction rates [39, 40]. Beyond chemistry, it has also been shown to influence superconductivity [41, 42], ferromagnetism [43], supramolecular self‐assembly [44], and long‐range energy transfer between donor–acceptor pairs [45, 46]. Cooperative vibrational strong coupling has been reported to increase the superconducting transition temperature of a superconductor, Rb_3_C_60_, which alone, cannot undergo strong coupling regime due to its weak transition dipole moment [41]. Rb_3_C_60_ is dispersed in a polystyrene matrix, having vibrational band overlap with the polymer, and thus undergoes CSC. Similarly, an enhancement in ferromagnetism is observed when YBa_2_Cu_3_O_7‐x_ molecule is blended in polystyrene [43]. The vibrational band of the polystyrene undergoes strong coupling with the cavity mode, and then the effects of vibro‐polaritonic states are transferred to the material by cooperativity. In these systems, a weak oscillator material is blended into the matrix of a strong oscillator. Recent studies suggest that the scattering parameters also change due to clustering when coupling solvent molecules to an infrared cavity mode [47]. These findings are in very good agreement with the theoretical observation of macroscopic condensation of Bogoliubov quasiparticles, which is one of many other notable findings in the literature [48].

Furthermore, CSC can be extended into the electronic strong coupling regime. A recent report demonstrated long‐range energy transfer between a donor–acceptor pair when the donor (MoS_2_) and acceptor (BRK) are cooperatively coupled [45]. To our surprise, the electron mobility of this system remains unchanged even if the donor–acceptor pairs are under CSC. This motivated us to try the effect of CSC in conventional organic semiconductors by varying the nature of the cooperative layers. To be more precise, here, we are exploring the idea of *polaritronics* [49, 50] under CSC conditions. To execute the idea, we considered an organic semiconductor—PDI (perylenediimide dye) as an electrically active layer and embedded inside the metal oxide semiconductor field‐effect transistor (MOSFET)‐based Fabry–Pérot (FP) cavity, along with another highly absorbing dye (Rhodamine‐B (RhB) or Perylene Red (Per‐Red)) [51, 52, 53, 54]. A large enhancement in the electron mobility of the PDI thin film is observed under CSC with the ionic RhB dye. On the other hand, the electrical properties of PDI remained unchanged under CSC with neutral Per‐Red dye. We explored this by comparing the band structure of the system by correlating both optical and electrical outputs. A mirrorless MOSFET configuration also shows a similar behavior under ON‐resonance coupling conditions. Our experiments demonstrated that the observed enhancement is due to the ionic nature of the RhB dye, which facilitates charge transport in PDI when the two are placed in direct contact. These effects are more pronounced when the CSC conditions are improved. This study opens a new pathway for tailoring the band structure of organic semiconductors by CSC.

## 2 Results and Discussion

MOSFET devices consisting of prepatterned gold electrodes interdigitated on a 90 nm SiO_2_‐coated silicon substrate are utilized for this study. Thin films are prepared by solution processing, using spin coating various solutions of active and inactive layers. RhB and Per‐Red thin films were prepared in PMMA/PVA polymers and are electrically nonconducting. PDI thickness is chosen (15 nm) in such a way that it cannot solely enter strong coupling regime. Two highly absorbing organic dye molecules chosen for CSC are: an ionic dye (RhB), and a neutral dye (Per‐Red). The molecular structures of these dyes are shown in Figure 1a. Thin films of PDI (0.1% in chloroform), RhB‐PMMA (4%–8% in acetonitrile, mixed in 1:1 ratio), Per‐Red/PMMA (8%–6% in anisole, mixed in 1:1 ratio) were coated on glass substrates for absorption measurements. The detailed preparation procedure for these solutions is described in the supporting information. The UV‐visible absorption spectra of 15 nm thick PDI, 270 nm thick RhB‐PMMA, 150 nm thick Per‐Red/PMMA is given in Figure 1b. The multiple peaks observed in the absorbance spectra of the PDI, RhB, and Per Red thin films originate from vibronic progression associated with the electronic transitions of the dye molecules. The thickness of the coated layers is determined by fitting absorption spectra in TMM. Both (RhB and Per‐Red) have very strong absorption bands overlapping with the PDI, specifically near 565 nm (shaded region). CSC can be achieved in the presence of the high oscillator (many molecules), and further, the electrical properties of the weak oscillator (PDI molecules, small in number) are probed. Figure 1c demonstrate the formation of two new eigenstates (upper polaritonic state (P^+^) and lower polaritonic state (P^−^)) under CSC when both the molecules are coupled to the same cavity mode. The schematic for MOSFET–Fabry–Pérot cavity devices utilized in this study is given in Figure 1d.

**FIGURE 1:**
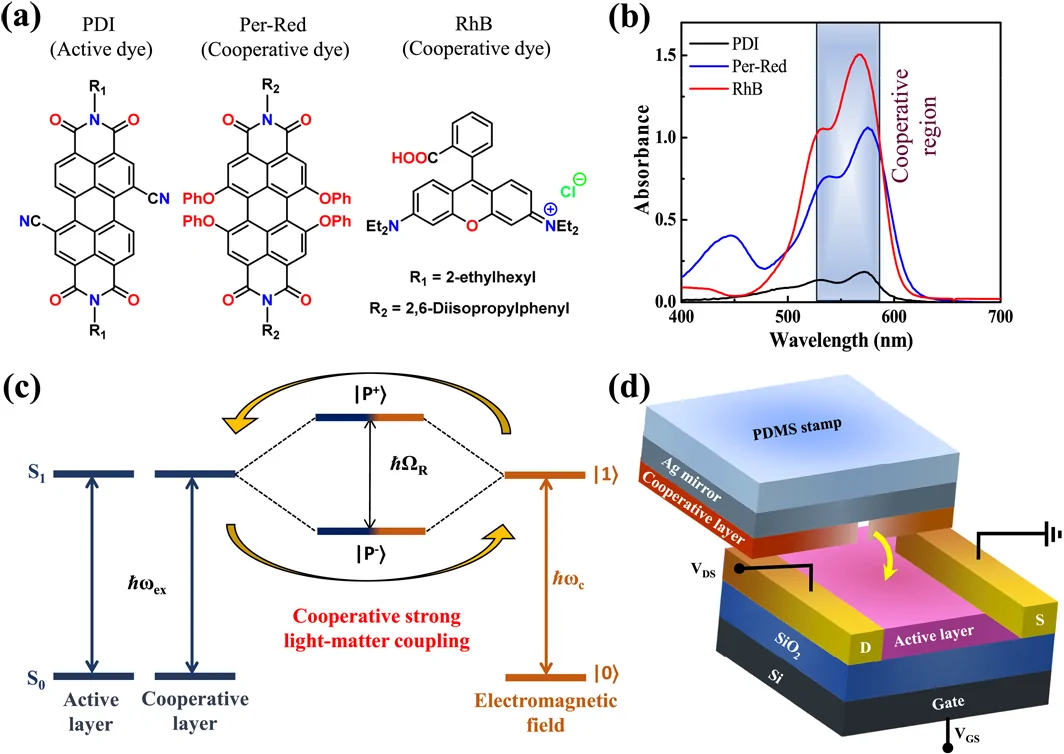
Molecular systems used for CSC experiments. (a) Molecular structure of PDI (active layer); Perylene‐Red, and Rhodamine‐B dyes (cooperative layers). (b) Absorption spectra of a thin film of each dye molecule. (c) Energy‐level diagram illustrating cooperative strong light–matter interaction. (d) Schematic for MOSFET‐FP cavity configuration used for CSC experiments.

Thin films of PDI were prepared by spin coating 0.1% PDI solution on MOSFET devices, and then were annealed at 100°C for half an hour in an inert atmosphere. This is considered as a noncavity. Reflectance spectra of PDI noncavity showed a broad leaky mode formation at lower energy (1.8 eV) than molecular absorption as shown in Supporting Information S1: Figure S1a. This leaky mode, arising from the refractive index contrast between the multilayers (PDI/SiO_2_/Si), has a poor quality factor of 2.1, as observed in our previous studies [14]. This mode is too leaky to get coupled to PDI absorption, showing minimal influence on electrical response. The thickness of the PDI thin film is maintained at a constant value (15 nm) for all experiments to ensure uniformity. All electrical measurements are performed in a dark, inert environment, with oxygen and water levels at the ppm level. Thickness and cavity mode positions were predicted by fitting experimental spectra using transfer matrix method (TMM).

We explored the mirrorless MOSFET system, building on our previous work [14]. For this, RhB (in PMMA) is spin‐coated at different speeds on a PDMS stamp and adhered on top of the PDI‐coated MOSFET device (similar to that shown in Figure 1d, but the Ag‐mirror is absent). The details of sample preparation are given in Supporting Information S1: Section S3. The effective refractive indices of various layers are taken as 1.47 (PDMS), 1.9 (RhB‐PMMA), 1.8 (PDI), 1.5 (SiO_2_), and 4 (Si). The multilayer configuration is shown in the inset of Figure 2c. The position of the leaky mode is tuned by changing the thickness of the RhB‐PMMA layer. The normal incidence reflectance spectra for three samples of varying thickness of RhB‐PMMA layer are shown in Supporting Information S1: Figure S1b. Here, the mirrorless configuration exhibits signatures of light–matter interaction; however, the hybrid states are not distinctly resolved due to the relatively weak coupling strength and the inhomogeneously broadened absorption line shape of the organic semiconductor. This is evident from the angle‐dependent dispersion experiments, as well as the TMM fitting (Figure 2a). Electrical measurements for a nearly ON‐resonant sample is shown in Figure 2b. The enhancement in PDI mobility in the presence of RhB relative to PDI‐only systems is plotted against leaky mode position in Figure 2c. This showed that charge transport in the PDI thin film is facilitated even under weak light–matter interaction with RhB. The detailed data on the mobility variation of PDI with mode tuning in the mirrorless configuration are provided in Supporting Information S1: Table S1. A slight enhancement in mobility, even at OFF‐resonance, is also observed here. Replacing a RhB‐PMMA layer with a Per‐Red/PMMA layer does not show leaky‐mode formation due to the lower effective refractive index (1.6). No clear signature of light–matter states is observed, as evident from the experimental normal incidence spectra of different samples of the PDI‐Per‐Red system shown in Supporting Information S1: Figure S1c. This is supported by the electrical measurements, which show negligible variation in PDI mobility when Per‐Red is placed on top, as shown in Supporting Information S1: Figure S2. The extracted mobility data for three samples are presented in Supporting Information S1: Table S2. This suggests that the leaky modes formed in a mirrorless MOSFET configuration can also affect electron mobility through cooperative light–matter interactions, even when the coupling conditions are weak.

**FIGURE 2:**
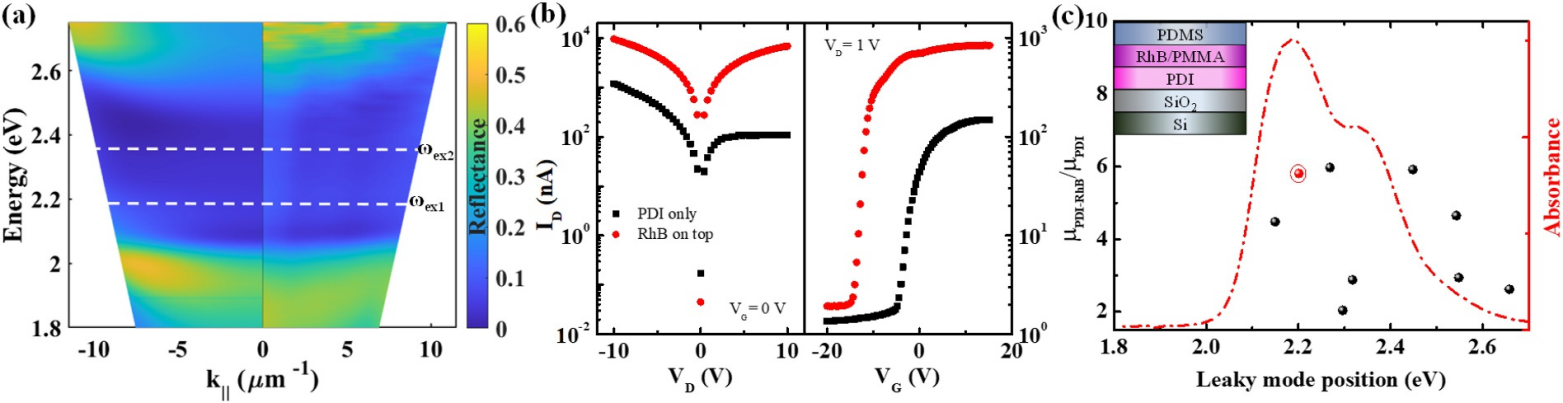
CSC with RhB in mirrorless MOSFET devices. (a) Optical response: Angle‐dependent spectra for mirrorless system with RhB as obtained from TMM (left) and experiment (right). Dash/dot lines serve as guides to the eye for polaritonic branches (yellow) and molecular absorption (white). (b) Electrical response: Output (left panel) and transfer characteristics (right panel) for PDI only (black), and ON‐resonance RhB (red) system. (c) Electron mobility ratio versus empty mode position showing enhancement under CSC. The inset shows a multilayer configuration of a mirrorless system. The electrical response shown in (b) corresponds to the red circled data point in (c).

Furthermore, to gain more insight into the nature of CSC, we studied MOSFET‐FP cavities. To begin with, the weak oscillator (PDI) is examined, and its optical and electrical characteristics are studied in a MOSFET‐FP cavity configuration (Figure 1d). Then, CSC with RhB or Per‐Red dyes are further tested in the same configuration and compared. Each sample is analyzed both optically and electrically, and its results are further correlated with TMM. PDI‐only MOSFET‐FP cavities comprise Ag/PMMA/PDI/SiO_2_/Si configuration. Cavity samples are prepared by adhering the PMMA/Ag‐coated PDMS stamps on top of the PDI‐coated MOSFET device. The full width half maxima (FWHM) for empty cavity ($\Gamma_{\text{cav}}$) and molecular absorption ($\Gamma_{\text{mol}}$) is 0.245 and 0.323 eV, respectively. The confined cavity mode exhibits an improved Q‐factor (8.9 at 2.19 eV), as observed from the TMM‐predicted spectra in Supporting Information S1: Figure S3a. The reflectance spectrum exhibits a modified cavity resonance near the molecular absorption region, indicating light–matter interaction. However, the splitting is not fully resolved, suggesting weak‐to‐intermediate coupling rather than pronounced strong coupling. The PMMA spacer thickness controls the cavity mode position and can be tuned from ON to OFF‐resonance conditions. The experimental reflectance spectra of different cavities with varying PMMA thickness are shown in Supporting Information S1: Figure S3b. TMM simulated dispersion for the empty cavity, and the ON‐resonant cavity is shown in Supporting Information S1: Figure S4. The electric field intensity distribution (∣E∣^2^) was calculated using the Transfer Matrix Method (TMM) based on the optical parameters of the multilayer structure. It splits into two new positions, supporting the observations from the dispersion plot (Supporting Information S1: Figure S4c,d). It also confirmed that the second‐order cavity mode is coupled to the active layer, which is positioned at the antinode. Electron mobility is extracted from transfer characteristics (*V*_*D*_ = 1 V) in the linear regime for each cavity and its corresponding noncavity samples (see the Experimental section). Electrical response of an ON‐resonant and one of the OFF‐resonant cavities with their corresponding noncavities is shown in Supporting Information S1: Figure S5. Electron mobility data obtained for each noncavity and corresponding cavity, along with empty cavity mode positions, are given in Supporting Information S1: Table S3 and Figure S6. Electron mobility remains almost the same from ON to OFF‐resonances, possibly because the system is at the onset of the strong coupling. In other words, coupling is not enough to affect the charge transport properties of the material under these conditions.

As noted above, the electrical properties of the PDI layer, approximately 15 nm thick, remain unchanged within a MOSFET‐FP cavity due to its weak coupling strength. Placing another highly absorbing dye (RhB or Per‐Red), along with PDI, can bring the system into a strong coupling regime. To achieve this, PMMA is replaced by RhB/PMMA and Per‐Red/PMMA for CSC studies. For cavity preparation, a solution of RhB in PMMA (in acetonitrile) or Per‐Red in PMMA (in anisole) is prepared and spin‐coated on an Ag‐sputtered PDMS stamp. The details of sample preparation are given in Supporting Information S1: Section S3. The cavity configuration comprises Ag/(RhB or Per‐Red)‐PMMA/PDI/SiO_2_/Si. More than a dozen independent samples with systematically varied mode positions were fabricated and characterized using both optical and electrical measurements to reach a final conclusion. The ON‐resonance condition is achieved when the cavity mode overlaps with the molecular transition (*ω*_ex1_). The corresponding effect of CSC is visible in the electrical measurements, where variation in electron mobility follows the absorption lineshape.

The excitonic resonances are structured due to the vibronic progression of the electronic transition, as shown in Figure 3a. We mapped the first (0, 0) and the second (0, 1) vibronic states to *ω*_ex1_ and *ω*_ex2_, respectively. However, these states are not well‐resolved in the system. Therefore, a two‐coupled oscillator model is employed here, and the dominant excitonic peak (*ω*_ex1_) is taken as the ON‐resonance position. Cavity mode tuning from ON to OFF‐resonance is performed by varying the thickness of RhB or Per‐Red layer. Rabi splitting is calculated from reflectance spectra from the difference between upper and lower polaritonic branches of an ON‐resonance sample. CSC with RhB dye gave a Rabi splitting energy of around 0.546 eV, which is more than the average of the FWHM of the empty cavity (0.227 eV) and molecular absorption (0.341 eV) (Figure 3a). The confined cavity mode at ON‐resonance gives a Q‐factor of 9.6. Similarly, CSC of Per‐Red cavities gives Rabi splitting energy (0.444 eV) more than the average of the FWHM of the empty cavity (0.249 eV) and molecular absorption (0.318 eV), confirming that both systems enter into strong coupling regime. The cavity mode for Per‐Red cavities gives a Q‐factor of 8.7. Experimental angle‐dependent studies (obtained from back focal imaging) for both dyes showed good agreement with TMM dispersion, clearly confirming the formation of polaritonic branches (Figure 3b,c). Further, the electric field intensity (|E|^2^) profile for both the systems for the empty cavity as well as the ON‐resonant cavity confirms the coupling to the second‐order cavity mode (*λ*). It also showed that the absorber dyes are positioned at the field maxima, therefore experience maximum electric field, as shown in Figure 3d.

**FIGURE 3:**
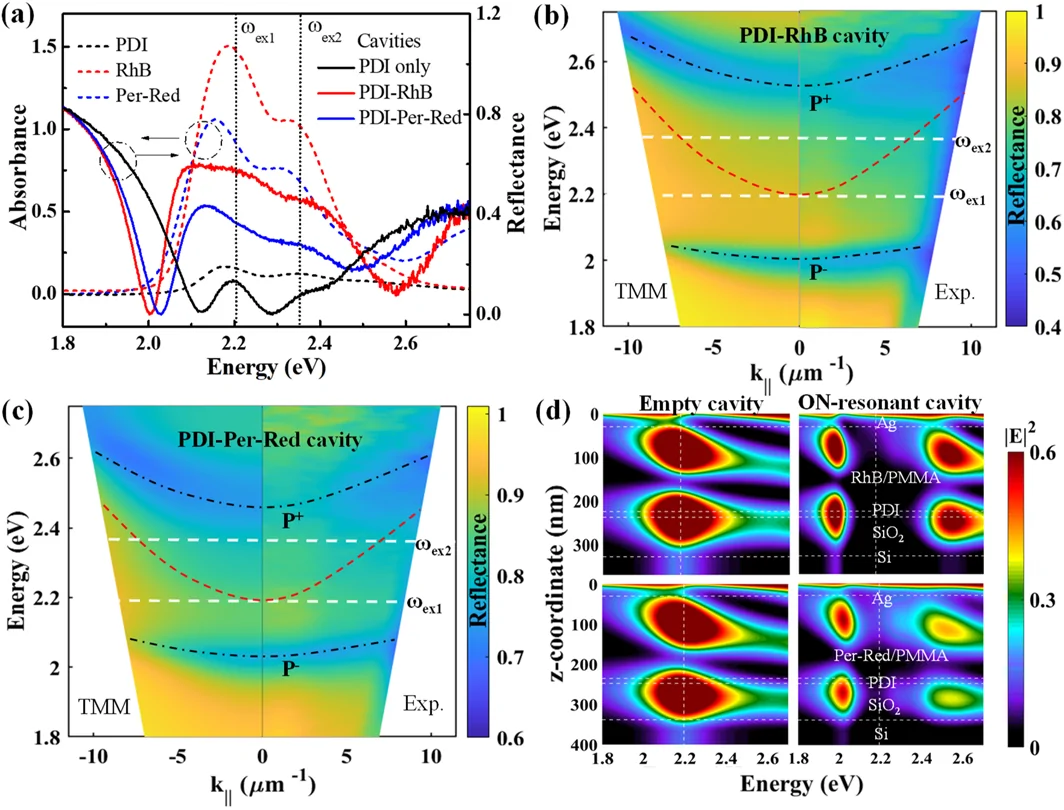
CSC in MOSFET‐FP cavity system—Optical output. (a) Normal incidence reflectance spectra for ON‐resonant cavities with RhB (red) and Per‐Red (blue). Dashed lines represent the absorption spectra of PDI (black), RhB (red), and Per‐Red (blue) thin films coated on glass substrates, respectively. Angle‐dependent spectra for (b) RhB cavity and (c) Per‐Red cavity as obtained from TMM (left panel) and experiment (right panel). Dashed lines are a guide to the eye. (d) Corresponding electric field distribution (|E|^2^) profiles for RhB cavity (upper panel), and Per‐Red cavity (lower panel) at ON‐resonance conditions.

Electrical measurements are performed on each noncavity sample (PDI‐only) before cavity preparation, and serve as a control experiment. An enhancement in the electron mobility is observed at ON‐resonance under CSC in RhB cavities when RhB layer is in direct contact with the active layer (Figure 4a,b). Electron mobility enhancement is also observed at OFF‐resonant conditions, as evidenced by the output and transfer characteristics (Figure 4a). Electron mobility data extracted from more than a dozen of independent MOSFET‐FP cavity devices from ON to OFF‐resonance is given in Supporting Information S1: Table S4. Please note that the enhancement is more at the ON‐resonance, and it follows its molecular absorption profile (Figure 4b). Inset of Figure 4b shows the multilayer cavity configuration when RhB is in direct contact with the active layer. Furthermore, we explored the RhB layer separated from the active layer by a PMMA spacer in the cavity. The multilayer configuration is shown in the inset of Figure 4d. Here, RhB is dissolved in a PVA solution (in water) and spin‐coated onto an Ag mirror‐coated PDMS stamp. A PMMA layer (prepared in anisole) is coated on top and then adhered to a PDI‐coated MOSFET device to complete the cavity configuration. Experiments are performed by taking thin (40 nm) and thick (230 nm) PMMA spacers. The details of sample preparation are given in Supporting Information S1: Section S3. For a thin PMMA spacer of 40 nm thickness, coupling to the second order cavity mode (Q‐factor of 8.8) gives a Rabi splitting energy of 0.560 eV, which is more than the average of FWHM of cavity mode (0.250 eV) and the molecular transition (0.341 eV), following the strong coupling criteria (as shown in TMM predicted spectra in Supporting Information S1: Figure S7a). The cavity tuning spectra obtained by changing the thickness of the RhB‐PVA layer are shown in Supporting Information S1: Figure S7b. TMM predicted dispersion and field distribution profile (|E|^2^) also supports the observation (Supporting Information S1: Figure S7c,d). Enhancement in electron mobility is observed at ON‐resonance, as shown in the plot of mobility variation with cavity mode position in Supporting Information S1: Figure S8. Data on electron mobility as a function of cavity tuning are provided in Supporting Information S1: Table S5.

**FIGURE 4:**
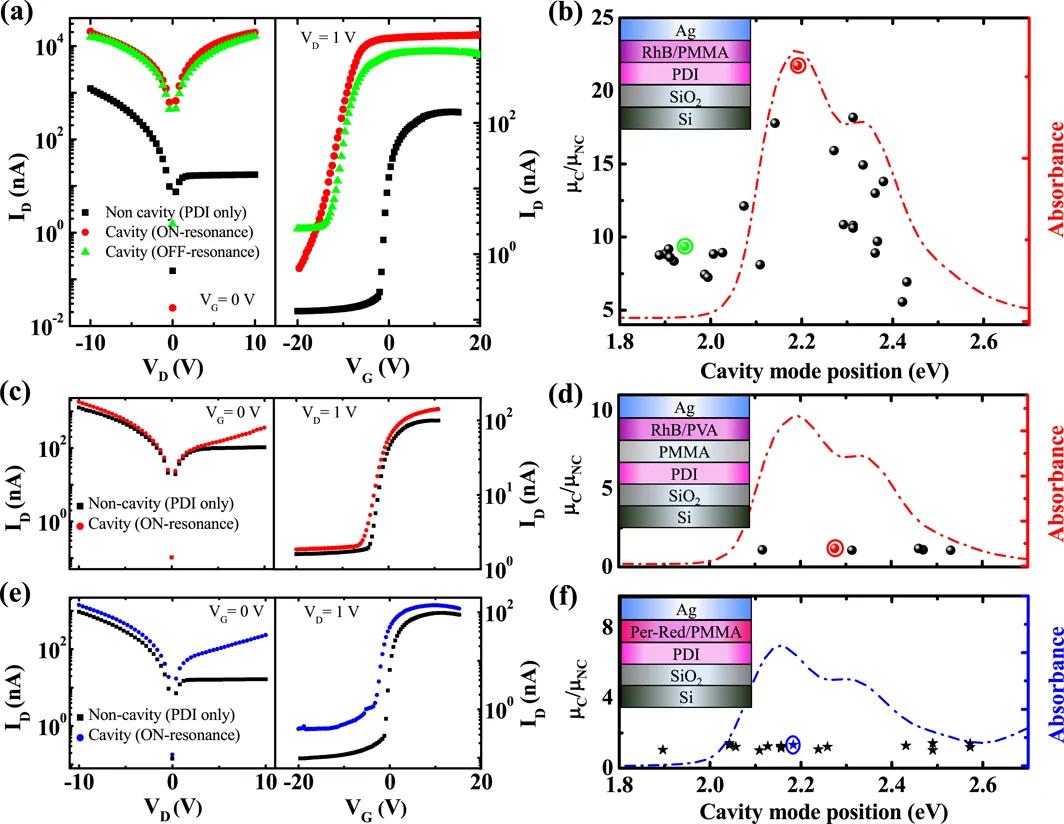
CSC in MOSFET‐FP cavity system—Electrical output. (a) Output (left panel) and transfer characteristics (right panel) for PDI noncavity (black), OFF (green), and ON‐resonant cavity (red) when RhB is in direct contact with PDI. (b) Corresponding electron mobility versus empty cavity mode position, showing enhancement under CSC. The inset shows a multilayer configuration for a cavity system. The electrical response shown in (a) is for the red and green circled data points in (b). (c) Output (left panel) and transfer characteristics (right panel) for PDI noncavity (black) and cavity (red) when RhB is separated from PDI by using a thick spacer (230 nm). (d) Corresponding electron mobility versus empty cavity mode position, showing a negligible effect of CSC. The inset shows a multilayer configuration for the cavity system used. The electrical response shown in (c) corresponds to the red‐circled data point in (d). (e) Output (left panel) and transfer characteristics (right panel) for PDI noncavity (black) and ON‐resonant cavity (blue) under CSC when Per‐Red is in direct contact with PDI. (f) Electron mobility versus empty cavity mode position under CSC with Per‐Red showing negligible enhancement. The inset shows a multilayer configuration of Per‐Red cavities. The electrical response shown in (e) corresponds to the blue‐circled data point in (f). Dash/dot lines in figures (b), (d) and (f) show the absorption profile of the corresponding uncoupled molecules.

Another set of experiments is performed by increasing the PMMA spacer thickness to 230 nm. Here, coupling to the third‐order cavity mode (Q‐factor of 12.7) gives a Rabi splitting of 0.460 eV. This is also greater than the average of the FWHM of the empty cavity (0.169 eV) and the molecular absorption (0.341 eV), and again, following the strong coupling criteria (Supporting Information S1: Figure S9a). Experimental cavity tuning spectra obtained by changing the thickness of the RhB‐PVA layer is shown in Supporting Information S1: Figure S9b. This is further confirmed by the dispersion of polaritonic states and cavity field distribution profile, suggesting that the third‐order cavity mode (3λ/2) is coupled to the molecular transition (Supporting Information S1: Figure S9c,d). Coupling to the *λ*‐mode is not possible since the cavity thickness is high due to the thick PMMA spacer. Electron mobility data for cavity and noncavity samples with the corresponding empty mode position are given in Supporting Information S1: Table S6. Negligible change in the electron mobility at the ON‐resonance occurs when RhB and PDI are separated by a very thick spacer (Figure 4c,d). The above results confirm that PDI mobility is altered only when both molecules (PDI and RhB) are in close contact. There is a possibility of interfacial charge transfer (ICT) between RhB and the PDI layer. This effect may be getting enhanced further when the molecules are placed inside the cavity, facilitated by CSC between the PDI and RhB molecules due to the formation of new polaritonic states.

Further replacing RhB with a neutral dye (Per‐Red) results in negligible variation in the electron mobility of PDI at the ON‐resonance condition when both layers are placed in direct contact under CSC (Figure 4e). The electron mobility data extracted for PDI noncavities and the corresponding ON‐to‐OFF resonance cavities for the Per‐Red system are given in Supporting Information S1: Table S7. A negligible change is observed in the electron mobility of the PDI layer versus cavity mode positions with Per‐Red from ON to OFF‐resonance conditions (Figure 4f). Furthermore, thin RhB‐PMMA films with a very low RhB concentration are prepared to prevent the formation of leaky modes in the mirrorless configuration (Supporting Information S1: Section S4). This can be achieved by lowering the refractive index contrast of the cooperative layer. A RhB‐PMMA noncavity, made by RhB‐PMMA‐coated PDMS, is the best control for such a comparison. Optical output of the sample‐1 and sample‐2 noncavity devices made by adhering RhB‐PMMA thin films on PDI, which clarifies the absence of any leaky modes (Supporting Information S1: Figure S10a). The electrical measurements of both samples, compared to their corresponding PDI‐only noncavities, showed an enhancement in electron mobility (Supporting Information S1: Figures S10b,c). This clarifies that some ICT occurs at the RhB‐PDI interface, which is responsible for the enhancement in mobility, even in the absence of strong coupling. The complete analysis of charge‐transport enhancement in cavity and noncavity systems, along with its dependence on spacer thickness, is shown in Supporting Information S1: Figure S11. Charge transfer between RhB and PDI fundamentally requires an interface, where RhB acts not as the conducting layer but rather facilitates electron injection into the active PDI layer through interfacial interactions, consistent with earlier reports [55]. Our control experiments show that although direct contact between RhB and PDI enhances conductivity even in noncavity structures, inserting a spacer suppresses this effect. Under cavity conditions, cooperative strong coupling amplifies the interfacial charge‐transfer process and sustains it over small spacer thicknesses, although the effect weakens beyond ∼40 nm (Supporting Information S1: Figure S11). Therefore, interfacial charge transfer and cooperative strong coupling operate synergistically. We attempted to probe for any ICT state using various spectroscopic techniques. The thin‐film configuration does not provide direct evidence of the ICT state, potentially limiting our ability to comment further on the nature of the interaction. Furthermore, we observed that the thin films themselves do not alter their physical structure. Instead, we believe that the CSC creates a more favorable electronic environment for ICT, thereby facilitating charge transport. The results concluded from all the above studies are shown in Table 1.

**TABLE 1:** Summary of the results obtained in CSC experiments (electron mobility in cavity systems is compared to the noncavity systems).

Sr. No.	Cavity system	Molecular FWHM (eV)	Empty cavity FWHM (eV)	Rabi splitting energy (eV)	Electron mobility
1	Mirrorless RhB	—	—	—	Enhancement
2	Mirrorless Per‐Red	—	—	—	No change
3	PDI‐only cavity	0.323	0.245	0.149	No change
4	Cavity RhB (direct contact)	0.341	0.227	0.546	Large enhancement
5	Cavity RhB (40 nm spacer)	0.341	0.250	0.560	Small enhancement
6	Cavity RhB (230 nm spacer)	0.341	0.169	0.417	No change
7	Cavity Per‐Red (direct contact)	0.318	0.249	0.444	No change
8	Noncavity RhB	—	—	—	Enhancement

To further understand the reasons behind electron mobility variation under CSC, variation in the effective mass of the lower polaritonic state is calculated from optical cavity tuning spectra using the Hopfield coefficient as discussed in Supporting Information S1: Section S5 (Supporting Information S1: Figure S12a,b). The lower polariton effective mass changes ∼10^−4^ with respect to uncoupled PDI as shown in Supporting Information S1: Figure S12c. To combine the optical and electrical results, we determined variation in Schottky barrier height (SBH) for both RhB and Per‐Red systems using the thermionic electron transport relation, as given in Equation (1) [56]:

(1) $$\Phi_{C}-\Phi_{NC}=\frac{kT}{e}\ln\left(\frac{I_{NC}}{I_{C}}\frac{m_{C}^{*}}{m_{ex}^{*}}\right)$$

Here, $\Phi_{NC}$ and $\Phi_{C}$ are SBH for noncavity (PDI‐only) and cavity (PDI in the presence of RhB or Per‐Red), respectively. $I_{NC}$ and $I_{C}$ is the drain current at a fixed biased voltage, $m_{ex}^{*}$ and $m_{C}^{*}$ are effective mass of the polaritonic state ($m_{LP}$ for P‐ state) and uncoupled PDI, respectively. Here we considered $m_{ex}^{*}$ as the bare electron mass. A relative variation in SBH for both RhB and Per‐Red is obtained, but a large decrease is obtained for the RhB case, as shown in Figure 5a. This suggests that CSC contributes to band tuning of the active layer, as represented schematically in Figure 5b.

**FIGURE 5:**
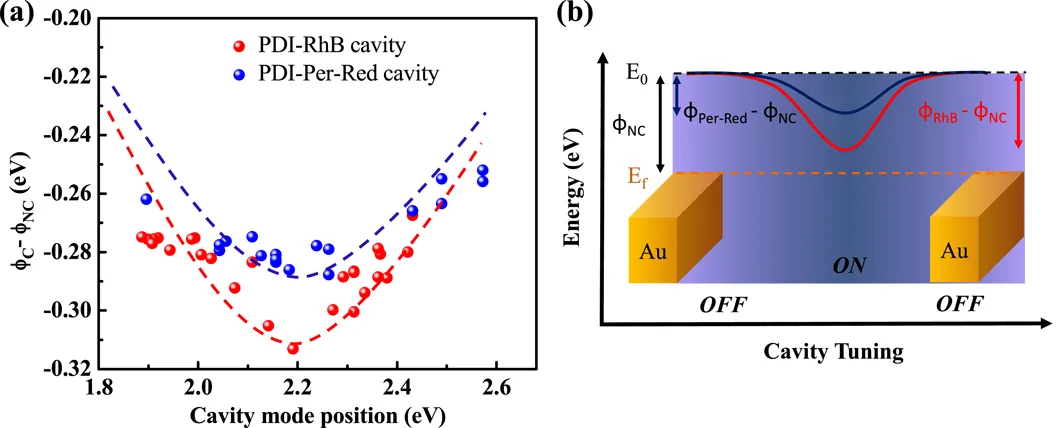
Tuning the band structure of organic semiconductors in a cavity. (a) Relative change in SBH w.r.t. noncavity (PDI only) under CSC with RhB (red) and Per‐Red (blue) systems. Dashed lines are a guide to the eye. (b) Schematic representation of SBH variation for both systems with cavity tuning.

The above calculation is the combined output of optical and electrical measurements done on a large number of independent devices. The resonance effect observed under CSC is reassuring, indicating that electron transport is enhanced, similar to the previous observation of direct strong coupling of either an electronic state [10, 13, 14] or a vibrational state [17]. There are several predicted mechanisms that include collective behavior, lowering the effective mass, and modification of the work function under strong coupling. Here, we employed the concept of work function modification to elucidate the nature of the interaction. The intricacies associated with the ICT channel under CSC are not clear to us; however, the results promisingly show that CSC can also be used to modify the electrical properties of a coupled system. Please note that polaritonic states (quasiparticles) are not the carriers in any of these cases; therefore, they will not directly influence carrier mobility. However, it may facilitate charge separation, thereby affecting carrier mobility. Several experimental studies have been conducted so far, supporting improved charge separation in strong coupled systems [25, 57, 58, 59]. On the other hand, the change in the electronic band structure, and therefore the variation of the work function, also plays a crucial role in electron mobility measurements. This is especially true for a bottom‐gated field‐effect transistor, in which the injection threshold depends solely on the SBH. Here, we propose a simple strategy to tailor the electrical properties of an organic semiconductor without chemically modifying the active layer using CSC experiments.

## 3 Conclusions

This work demonstrates the effect of CSC on the electron mobility of a weakly absorbing organic semiconductor in the presence of highly absorbing organic dye molecules. CSC is achieved by coupling with strong oscillators such as RhB or Per‐Red, as confirmed from optical studies. However, the effect of CSC on electron mobility is observed when the RhB layer is placed in direct contact with the PDI. Similar results are obtained when coupling to the leaky modes of a mirrorless MOSFET configuration as well. However, a feeble effect is observed due to weak light–matter interaction in the system. A comparative analysis indicates that the observed changes in electrical response correlate with the resonance condition. Our detailed studies, supported by various control experiments, suggest that direct interfacial charge transfer between RhB and PDI promotes electron mobility, which is further enhanced by cooperative strong coupling. In a nutshell, we leveraged interfacial effects and cooperative light–matter interactions to prepare the best‐performing next‐generation optoelectronic devices.

## 4 Experimental Section

### 4.1 Sample Preparation

Bottom‐gate‐bottom‐contact MOSFET devices consisting of highly doped n^++^‐silicon substrates coated with 90 nm SiO_2_ as a dielectric layer and prepatterned interdigitated Ti/Au electrodes of specific length (20 μm) and width (2 mm) (purchased from Fraunhofer‐IPMS, Germany), were used in this study. Prior to use, the substrates underwent ultrasonic cleaning in electronic‐grade acetone and isopropyl alcohol, and were then blow‐dried with high‐purity nitrogen. Almost the same thickness of PDI (15 nm) was used in all cases obtained by spin coating (using LABSPIN 6, Suss‐Microtech), 60 μL of 0.1% PDI solution in chloroform (filtered with PTFE filters of 0.45 μm diameter) at 750 rpm and acceleration of 4000 rpm/s for 60 s. Samples were postannealed for 30 min at 100°C under an inert atmosphere, to improve molecular alignment. All other coatings were done on a PDMS stamp, which then adhered to PDI‐coated MOSFET devices. This dry‐transfer approach was utilized to shield the underlying PDI layer from solvent‐induced degradation or contamination. The procedures for coating other layers on PDMS are given in Supporting Information S1.

### 4.2 Optical Studies

Static reflectance measurements were performed at room temperature using a Nikon Eclipse Ti_2_ inverted microscope equipped with a halogen lamp as the broadband light source. The reflected signal was collected through a 20X objective (NA = 0.45) and directed into a spectrometer (SpectraPro HRS‐300, Princeton Instruments). Spectral acquisition was performed using a charge‐coupled device (CCD) camera (Pylon 100BX), which was cryogenically cooled to −120°C with liquid nitrogen to minimize thermal noise.

### 4.3 Back‐Focal Plane Spectroscopy

Angle‐resolved reflection spectra were acquired using a 4f relay optical configuration incorporating a Fourier imaging scheme. Broadband illumination from a halogen lamp was focused onto the sample through a 100X microscope objective, which simultaneously collected the reflected light. The reflected signal was collimated by the tube lens and relayed to the spectrometer using two achromatic lenses (*f* = 100 mm). To access the angular (momentum‐space) information, an additional achromatic lens (*f* = 50 mm) was placed before the spectrometer entrance slit to image the back focal (Fourier) plane of the objective onto the CCD detector.

### 4.4 Electrical Studies

The electrical characteristics of the three‐terminal bottom‐gate, bottom‐contact MOSFET devices were measured inside a nitrogen‐filled glove box under inert conditions (O_2_ and H_2_O levels < 10 ppm) using a Cascade Microtech MB‐150 probe station and a Keithley 2636B source‐measure unit controlled via KickStart 488 software. Output characteristics (I_D_‐V_D_) were recorded by sweeping the drain voltage at fixed gate voltages, whereas transfer characteristics (*I*_*D*_–*V*_*G*_) were acquired in the linear regime at a fixed drain voltage of *V*_*D*_ = 1 V. The field‐effect mobility was extracted from the linear regime using the slope of the transfer characteristics according to Equation (2):

(2) $$\mu_{\text{linear}}={\left(\frac{dI_{D}}{dV_{G}}\right)}_{V_{D}}\frac{L}{WC_{i}V_{D}}$$

Here, L (length of the electrodes) = 20 μm, W (width of the electrodes) = 2 mm, $C_{i}$ is the capacitance of the gate dielectric given as, $C_{i}=\frac{\varepsilon \varepsilon_{0}}{d}$, Where $\varepsilon_{0}$ is vacuum permittivity, ε is the permittivity of the medium and d is the thickness of the dielectric layer. Also, $C_{i}$ = 3.84 × 10^−8^ F cm^−2^ (for 90 nm SiO_2_). These parameters are kept constant for all experiments.

## Author Contributions

**Kuljeet Kaur:** methodology, data curation, investigation, validation, formal analysis, writing – review and editing, writing – original draft, visualization. **Nitin Yadav:** data curation, formal analysis, writing – review and editing. **Shashi Kumari:** data curation, formal analysis, writing – review and editing. **Jino George:** conceptualization, supervision, funding acquisition, project administration, resources, writing – original draft, writing – review and editing.

## Funding

J. G. thank the ANRF‐core research grant (CRG/2023/001122).

## Conflicts of Interest

The authors declare no conflicts of interest.

## Supporting information


Supporting Information S1

## Data Availability

The data that support the findings of this study are available on request from the corresponding author. The data are not publicly available due to privacy or ethical restrictions.
