# Supplementary material for: Enhanced Transport in Organic Semiconductors Mediated Through Interfacial Charge Transfer and Cooperative Strong Coupling
Source: Nanophotonics. 2026 Jul 24;15(14):e70197. doi: 10.1002/nap2.70197 (PMC13642105; doi:10.1002/nap2.70197)
Supplement: Supplementary file 1 [file NAP2-15-e70197-s001.pdf]

Supporting Information

Enhanced Transport in Organic Semiconductors Mediated Through Interfacial Charge Transfer and Cooperative Strong Coupling

Kuljeet Kaur, Nitin Yadav, Shashi Kumari, Jino George*

Molecular Strong Coupling Lab, Department of Chemical Sciences, Indian Institute of Science Education and Research (IISER) Mohali, Punjab, India - 140306.

*E-mail: jgeorge@iisermohali.ac.in

Table of Contents

Section S1 Light-matter interaction in mirrorless systems ... 2

Section S2 PDI only cavity ... 5

Section S3 CSC with RhB or Per-Red ... 9

Section S4 RhB/PDI *versus* distance-dependence ... 13

Section S5 Hopfield coefficient, effective mass and SBH calculation ... 14

References ... 16

Section S1 Light-matter interaction in mirrorless systems

A thin PDI film is prepared on a Si/SiO_2-substrate (MOSFET device) by spin coating 60 µLof 0.1% PDI solution (w/w% in chloroform, filtered through a 0.45 µm pore size PTFE filter) at 750 rpm for 60 seconds onto the MOSFET substrates under inert conditions inside a glove box. After spin coating, the samples are heated for 30 minutes in the dark to remove residual solvent. All the non-cavities are prepared in a similar way. It gives a thickness of around 15 nm. The experimental reflectance spectra of a thin PDI film coated on a 90 nm SiO_2/Si-substrate are shown in Figure S1(a). A leaky mode at a higher wavelength appears due to the refractive index contrast between the Air/PDI/SiO_2/Si interfaces. This leaky mode has a poor quality factor, so effective coupling with molecular absorption cannot be obtained with this, as reported in our previous studies [1]. The quality of the leaky mode gets improved when RhB is placed on top of PDI, as shown in Figure S1(b). This is achieved because of the high refractive index contrast between the multilayers. Here, for sample preparation, a solution was prepared by mixing a 4% RhB solution (w/w% in acetonitrile) and an 8% PMMA solution (w/w% in acetonitrile) in a 1:1 ratio, then stirring for minutes. After filtering through a 0.45 µm pore-size PTFE filter, it is coated onto PDMS stamps, which are then adhered to the PDI-coated MOSFET devices. Three different samples, referred to as Sample-1, 2, and 3, are prepared in the (PDI+RhB) case, where the mode position is tuned by varying the thickness of the RhB-PMMA layer while keeping the PDI layer thickness fixed. The effect of coupling to the leaky mode is also reflected in electrical measurements, as discussed in the main text. The electrical data for the variation in electron mobility of PDI for cooperative strong coupling (CSC) with leaky mode in the presence of RhB is given in Table S1.

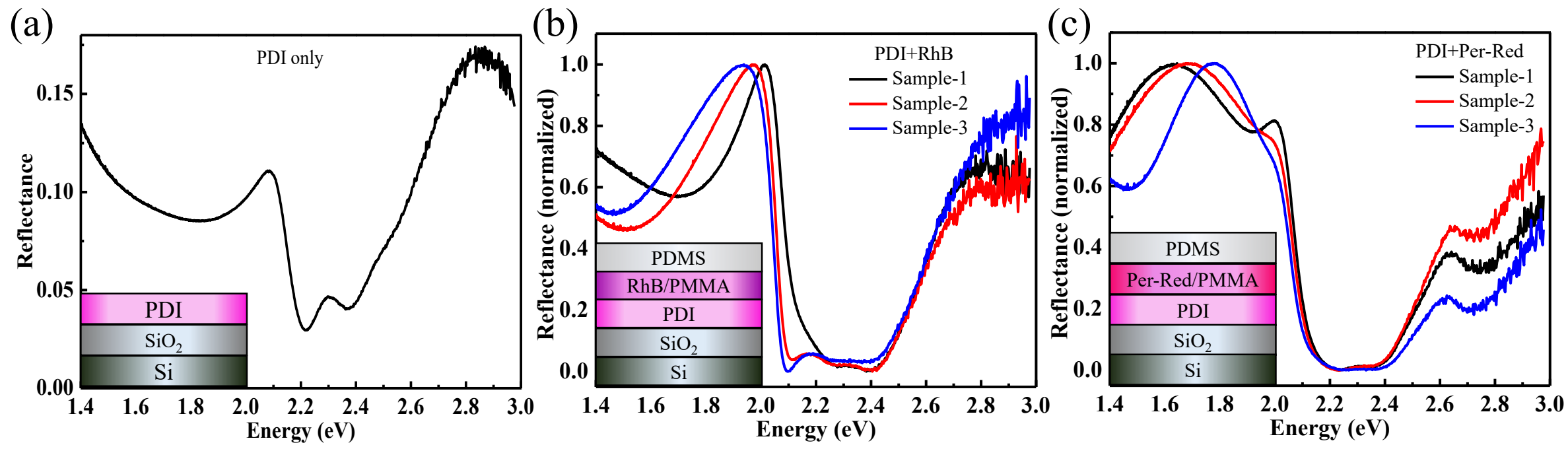

Figure S1. (a) Experimental normal incident spectra of PDI thin film coated on SiO_2/Si showing a leaky mode formation at lower energy due to the refractive index contrast between multilayers. (b) Experimental normal incident spectra of RhB placed on top of PDI reveal the formation of polaritonic branches. (c) Experimental normal incident spectra when Per-Red is placed on top of PDI. The inset in each figure shows the corresponding multilayer configuration.

Table S1: Cavity tuning data for CSC with leaky mode (RhB in direct contact with PDI).

Sr. No.	Empty cavity mode (nm)	μ_{NC} (10^{-3} $cm^2V^{-1}s^{-1}$)	μ_C (10^{-3} $cm^2V^{-1}s^{-1}$)	Mobility ratio (μ_C / μ_{NC})
1	467	4.580	12.014	2.623
2	487	3.841	11.317	2.946
3	488	3.690	17.153	4.649
4	506	3.909	23.133	5.918
5	535	3.536	10.196	2.883
6	540	3.402	6.951	2.043
7	547	4.028	24.061	5.973

8	563	3.437	19.968	5.809
9	570	4.039	18.094	4.479

Furthermore, three different samples of mirrorless systems are prepared in the presence of Per-Red labelled as (Sample-1,2,3 in (PDI+Per-Red)) to test under similar conditions. Here, 8% Per-Red (w/w% anisole) and 6% PMMA (w/w% in anisole) are prepared separately. These are mixed in a 1:1 ratio inside the glove box and kept stirring for minutes. After filtering the mixture through PTFE filters (0.45 μm pore size), it is spin-coated at different speeds onto the PDMS stamps, which are then adhered to the PDI-coated MOSFET devices. Here, strong coupling is not achieved because of the lower refractive index of Per-Red (effective refractive index ~ 1.6) as compared to RhB layers (Figure S1(c)). The electrical response of PDI in the presence of Per-Red shows negligible variation in electron mobility when measured on these samples (Figure S2). The mobility data obtained for three different samples are given in Table S2.

Table S2: Variation in electron mobility of PDI when placed in direct contact with Per-Red.

**Sample No.**	μ_{PDI} **(10^{-3} cm^{2}V^{-1}s^{-1})**	$\mu_{PDI+Per-Red}$ **(10^{-3} cm^{2}V^{-1}s^{-1})**	**Mobility ratio** ($\mu_{PDI+Per-Red}$ / μ_{PDI})
1	3.552	4.268	1.202
2	4.016	3.288	0.819
3	5.314	5.575	1.049

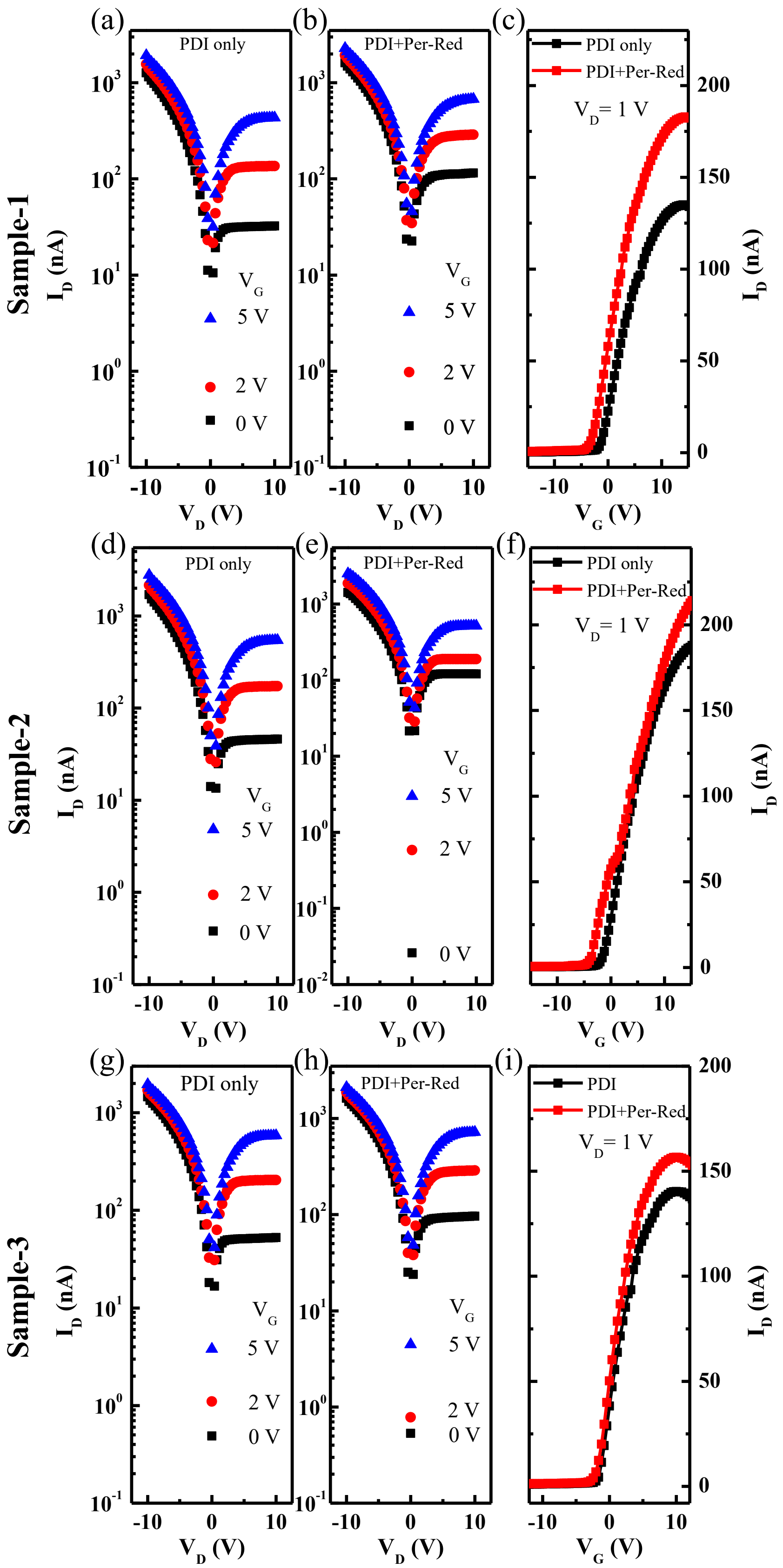

Figure S2. Electrical measurements of PDI in the presence of Per-Red. Figures (a), (d) and (g) show output characteristics of PDI coated on three different MOSFET devices. Figures (b), (e) and (h) represent output characteristics of these samples of PDI in the presence of Per-Red. Figures (e), (f), and (i) represent corresponding comparisons of transfer characteristics showing negligible variation.

Section S2 PDI only cavity

Here, the cavity structure comprises Si/SiO_2/PDI/PMMA/Ag mirror. A 30 nm silver mirror is sputtered on a PDMS stamp for cavity formation. 2-3% PMMA (w/w% in anisole) solution is spin-coated at different speeds on silver mirror-coated PDMS stamps. The PMMA spacer thickness controls the tuning of the cavity mode from ON to OFF-resonance. Each sample is analyzed optically and electrically, and further correlated with the TMM modelling. TMM predicted spectra at ON-resonance are shown in Figure S3(a). The ON-resonance condition is obtained for around 248 nm PMMA thickness for the second-order cavity mode. The experimental reflectance spectra of different cavities with varying PMMA thickness are shown in Figure S3(b).

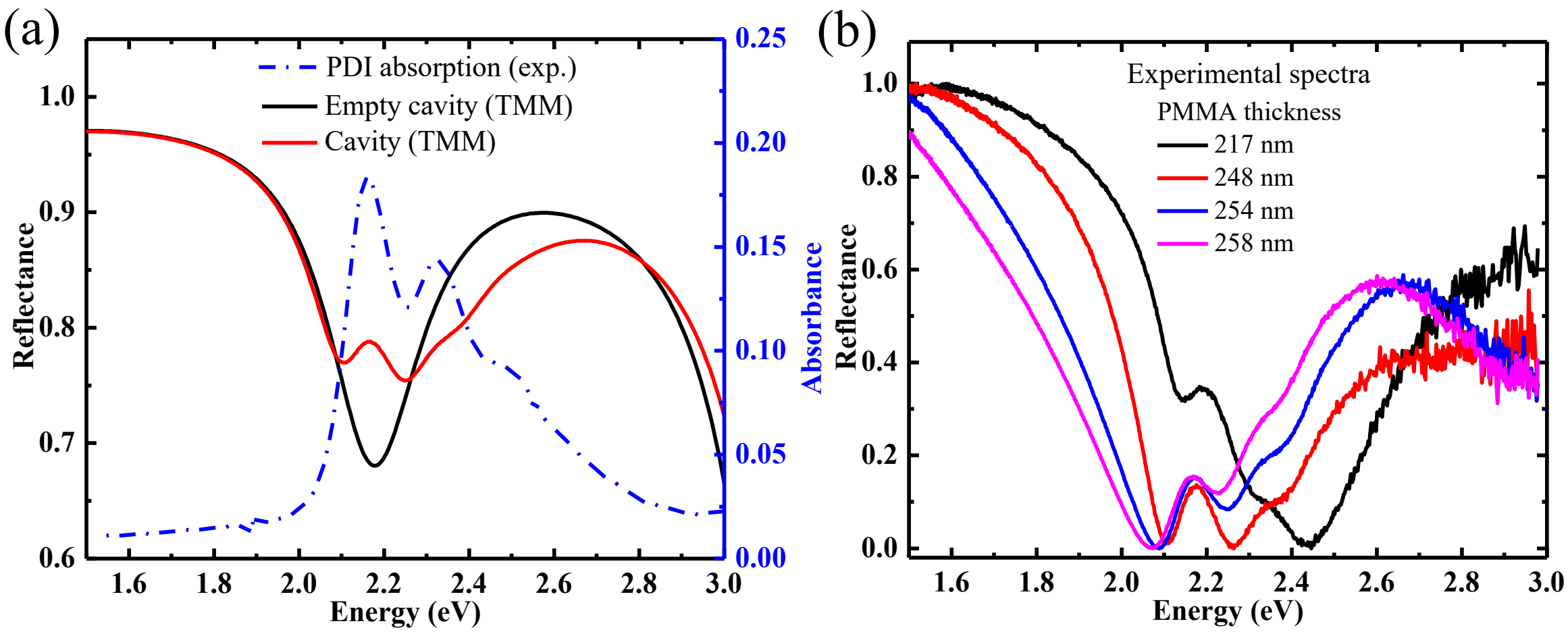

Figure S3. Optical characterization for PDI-only cavity. (a) TMM simulated spectra for the ON-resonant cavity (Ag (30 nm)/PMMA (248 nm)/PDI (15 nm)/SiO2 (90 nm)/Si) (red) and the corresponding empty cavity (black). (b) Experimental normal incidence spectra for cavity tuning obtained by varying the thickness of the PMMA layer.

The refractive index of the silver layer used in TMM analysis is taken from Johnson and Christy [2]. Refractive indices of PDMS, PMMA, SiO_2, and Si are obtained from the refractive index database [3]. Refractive index of PDI, RhB, and Per-Red layers is extracted using the Kramer-Kronig relation by fitting the corresponding absorption spectra. TMM simulated dispersion for the empty cavity, and the ON-resonant cavity is shown in Figure S4(a) and (b). A very small splitting can be clearly observed in cavity dispersion. Field distribution profile ($|E|^2$) for the empty cavity and the ON-resonant cavity (Figure S4(c) and (d)) confirmed that the second-order cavity mode is coupled to the active layer positioned at the antinode. Field intensity is also split into two new positions, supporting the observations from the dispersion plot. The electrical response of all the cavity devices is measured inside the glove box in the dark. The electron mobility extracted for each cavity system is compared to that of the corresponding non-cavity sample to reach a final conclusion.

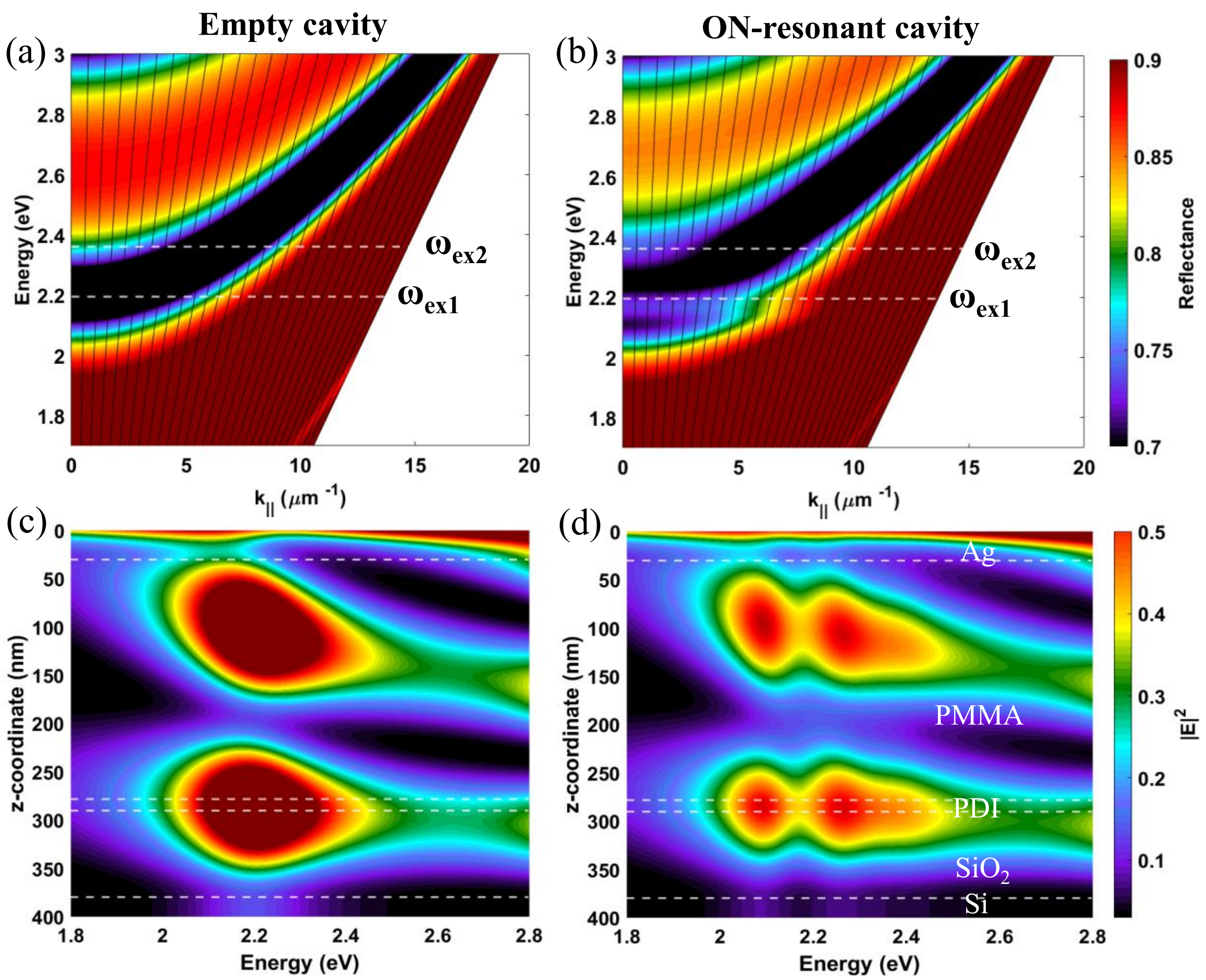

Figure S4. TMM simulated dispersion and field intensity distribution profile for PDI coupling. (a) and (b) are the dispersion plots for the empty cavity and the ON-resonant cavity, respectively. The white dashed line represents the molecular absorption. (c) and (d) represent the cavity electric field distribution profile ($|E|^2$) within multilayers for an empty cavity and the ON-resonant cavity for second-order mode coupling, showing the active layer positioned at the antinode. A branching of polaritonic states is clearly visible, but has not entered the strong coupling regime.

Figure S5 (a) and (b) shows the output characteristics of the non-cavity and the corresponding ON-resonant cavity device. Electron mobility is calculated from transfer characteristics at V_D = 1 V in the linear regime. Negligible variation in electron mobility is observed at the ON-resonance condition, as shown in the transfer characteristics in Figure S5(c). Further, the electrical response of an OFF-resonant sample (172 nm thick PMMA) also gave similar results, as shown in Figure S5 (d), (e) and (f). The thickness of each layer and the cavity mode position are obtained by fitting experimental spectra in the TMM simulation. Since all the samples are prepared independently, the thickness and electrical response of PDI non-cavities may vary slightly between samples.

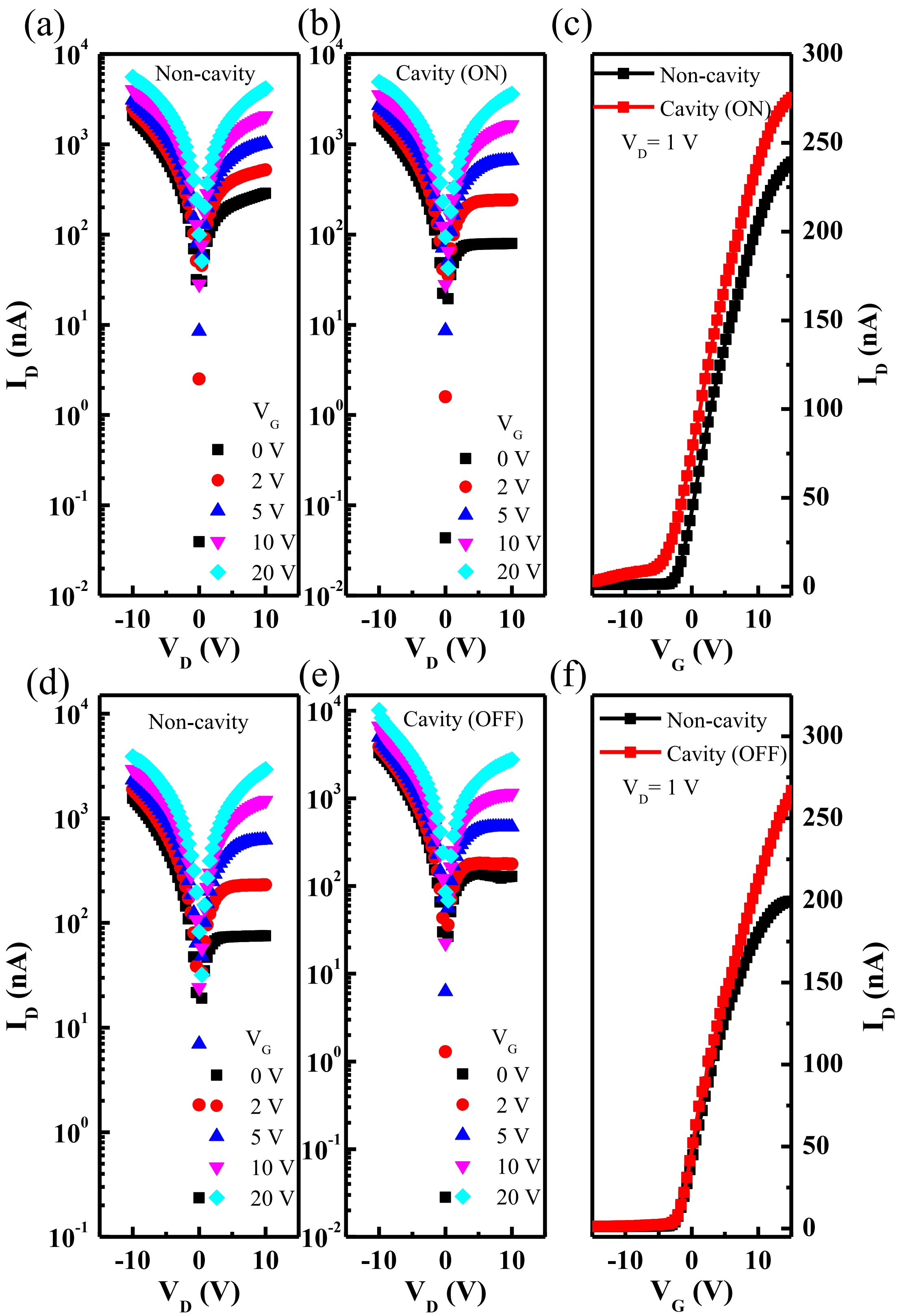

Figure S5. Electrical measurements of PDI-only cavity. The upper panel shows the output (a and b) and transfer characteristics (c) for the non-cavity and the corresponding ON-resonant cavity. Similarly, the lower panel shows the output (d and e) and the transfer characteristics (f) for the non-cavity and the corresponding OFF-resonant cavity. [Transfer characteristics are measured at V_D = 1 V]

The ratio of the electron mobility for each cavity (μ_C) to the corresponding non-cavity (μ_{NC}) is analyzed. The corresponding cavity mode position, along with the electron mobility ratio for all measured samples, is provided in Table S3. The mobility ratio *versus* cavity mode position is given in Figure S6. Electron mobility remains almost same from ON to OFF-resonance, possibly because the system is at the onset of the strong coupling. In other words, coupling is not enough to affect the charge transport properties of the material. This means that a PDI layer approximately 15 nm thick inside the cavity cannot reach strong coupling regime and therefore cannot induce any change in electrical response.

Table S3: Cavity tuning data for PDI-only cavity.

Sr. No.	Empty cavity mode (nm)	μ_{NC} (10^{-3} $cm^2V^{-1}s^{-1}$)	μ_C (10^{-3} $cm^2V^{-1}s^{-1}$)	Mobility ratio (μ_C / μ_{NC})
1	458	4.806	5.281	1.099
2	459	4.564	5.033	1.103
3	463	4.865	4.231	0.869
4	521	4.622	4.476	0.968
5	565	4.893	4.922	1.006
6	574	4.805	4.700	0.978
7	585	4.805	4.354	0.906
8	645	4.564	3.839	0.841
9	766	4.623	4.468	0.967

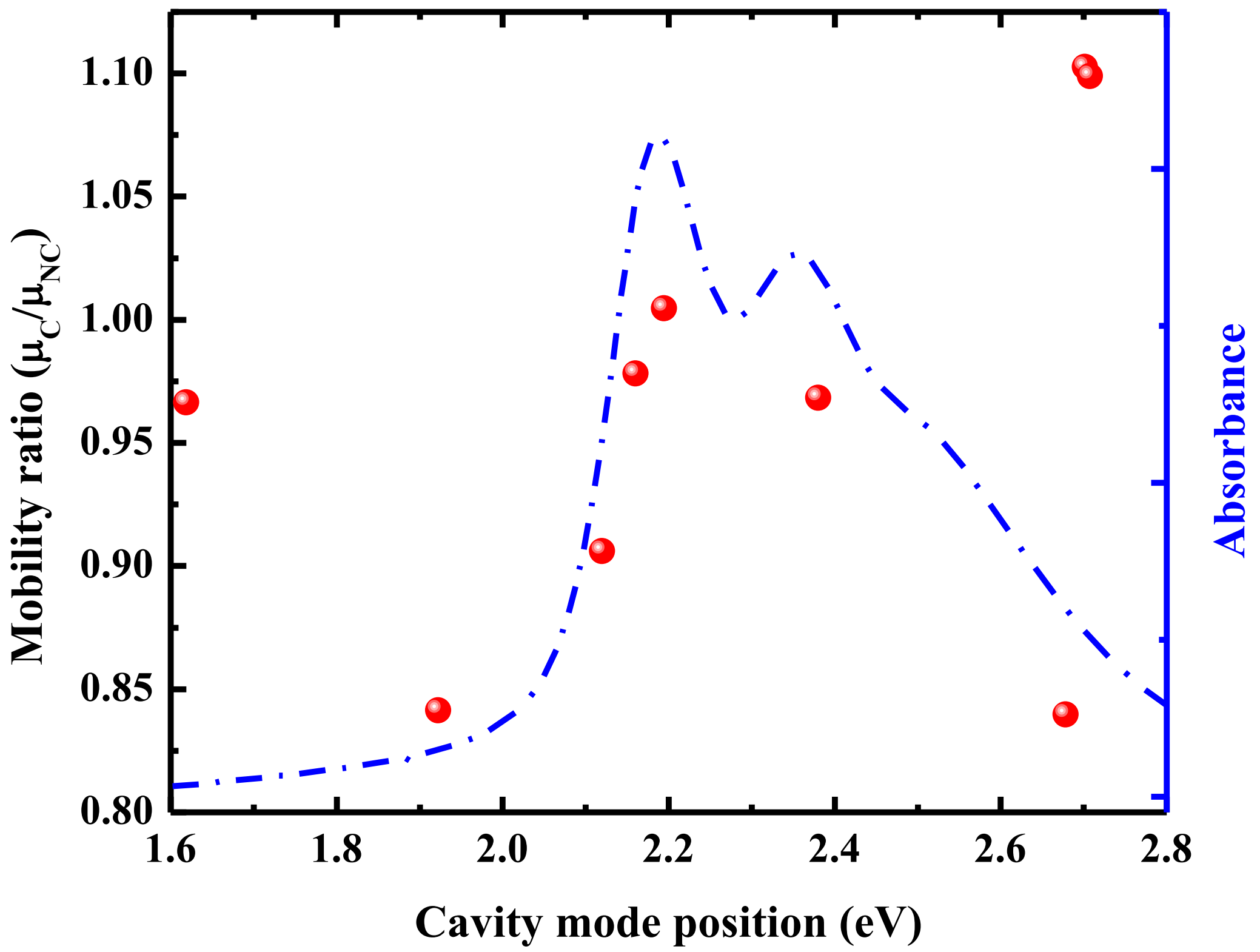

Figure S6. Variation in the electron mobility ratio with cavity mode position for second-order mode coupling in a PDI-only system.

Section S3 CSC with RhB or Per-Red

Sample preparation: To start with, a 30 nm thick Ag mirror is sputtered onto a PDMS stamp. For RhB cavities, 4% RhB solution (w/w% in acetonitrile) is mixed with the 8% PMMA (w/w% in acetonitrile) in a 1:1 ratio and filtered through PTFE filters. It is then coated on the thin silver mirror-sputtered PDMS stamps, which are subsequently adhered to the PDI-coated MOSFET devices to complete the cavity structure. 6%-8% Per-Red (w/w) solution prepared in anisole and mixed with 6% PMMA (w/w in anisole) solution in a 1:1 ratio under inert conditions. After filtering the mixture through PTFE filters (0.45 µm pore size), it is spin-coated at different speeds onto Ag-mirror/PDMS stamps to create the final cavity configuration for both optical and electrical studies. In the above cases, 100 µL of solution is used for spin coating at different speeds, with acceleration set to 4000 rpm/s for 60 sec. The extracted mobility variation data for CSC with RhB, along with the corresponding empty-cavity mode positions, are given in Table S4.

Table S4: Cavity tuning data for the CSC with RhB dye in direct contact with the PDI layer.

Sr. No.	Empty cavity mode (nm)	μ_{NC} (10^{-3} $cm^2V^{-1}s^{-1}$)	μ_C (10^{-3} $cm^2V^{-1}s^{-1}$)	Mobility ratio (μ_C / μ_{NC})
1	412	5.857	63.019	10.759
2	419	5.121	35.398	6.912
3	434	3.948	44.324	11.228
4	438	5.565	53.480	9.609
5	444	4.528	51.423	11.357
6	510	3.340	23.129	6.925
7	512	2.632	28.921	10.986
8	521	4.035	55.709	13.805
9	525	4.232	37.706	8.910
10	524	2.207	21.429	9.702
11	525	4.454	57.909	13.002
12	531	3.790	56.604	14.934
13	536	2.151	22.845	10.621
14	536	4.675	84.997	18.181
15	536	2.297	24.774	10.786
16	541	4.213	45.733	10.854
17	546	4.153	66.088	15.914
18	566	4.087	91.578	22.406
19	579	3.433	61.075	17.789
20	588	3.488	28.276	8.108
21	598	3.873	46.956	12.122
22	612	3.798	33.929	8.933
23	618	2.377	20.988	8.831
24	622	4.787	34.690	7.247
25	624	3.272	24.372	7.448
26	638	4.114	38.486	9.353
27	646	4.293	35.844	8.349
28	649	3.201	27.556	8.608
29	653	4.543	40.121	8.832
30	650	2.783	25.554	9.183
31	657	4.589	40.231	8.767

To check whether the same effect is observable when RhB is not in direct contact with PDI, experiments are performed by placing a PMMA spacer (40 nm and 230 nm) between PDI and RhB. The cavity configuration is shown in the inset of Figure S9(a). 3% RhB and 5% PVA (w/w%) solutions are prepared separately in distilled water. They are mixed in a 1:1 ratio and then filtered with a PTFE filter (pore size of 0.45 μm). This solution is spin-coated at various speeds onto an Ag-mirror sputtered PDMS stamp. Additionally, a PMMA layer is coated on top to separate the RhB-PVA layer from the active layer. For a thin PMMA layer of 40 nm, 2% PMMA solution (w/w% in anisole) is spin-coated at 4000 rpm, whereas for a thick PMMA layer of 230 nm, 6% PMMA solution (w/w% in anisole) is spin coated @2000 rpm. Since PVA is poorly soluble in anisole, RhB is mixed with PVA to form thin films. Thus, coating the PMMA solution (in anisole) on top has a very low chance of washing away the RhB-PVA layer. The cavity mode tuning is controlled by the thickness of the RhB-PVA layer. For the case where a 40 nm thick PMMA spacer is used, ON-resonance is with a 165 nm thick RhB-PVA layer. The normal incidence spectra obtained from TMM and the cavity configuration is shown in the inset of Figure S7(a). TMM predicted dispersion and field distribution profile ($|E|^2$) is shown in Figure S7(c) and (d). Experimental cavity tuning spectra for different RhB-PVA thicknesses are shown in Figure S7(b). An enhancement in electron mobility at ON-resonance is observed for a 40 nm thick spacer, as shown in Figure S8. The variation in electron mobility for both cavity and non-cavity structures with the cavity mode position is presented in Table S5.

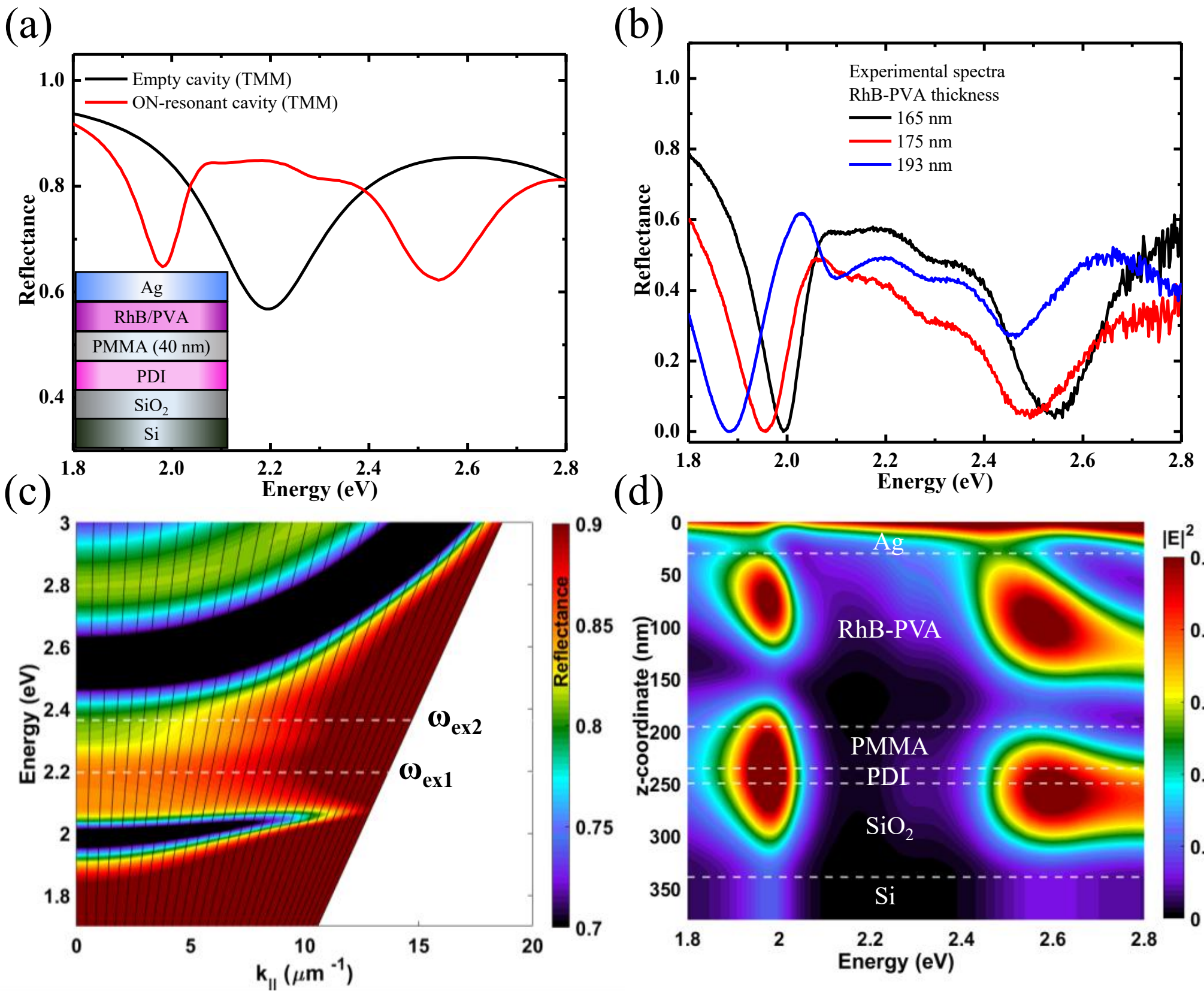

Figure S7. (a) TMM simulated spectra for the ON-resonant cavity (red) and the corresponding empty cavity spectra (black) when spacer thickness is 40 nm. The inset shows the multilayer structure used for the CSC experiment. (b) Experimental cavity tuning spectra obtained by varying the thicknesses of the RhB-PVA layer. (c) TMM simulated dispersion plot for the ON-resonant cavity for CSC of RhB from a second-order cavity mode. The white dashed line represents the molecular absorption band. (d) The corresponding electric field distribution ($|E|^2$) for the ON-resonant cavity within the multilayers.

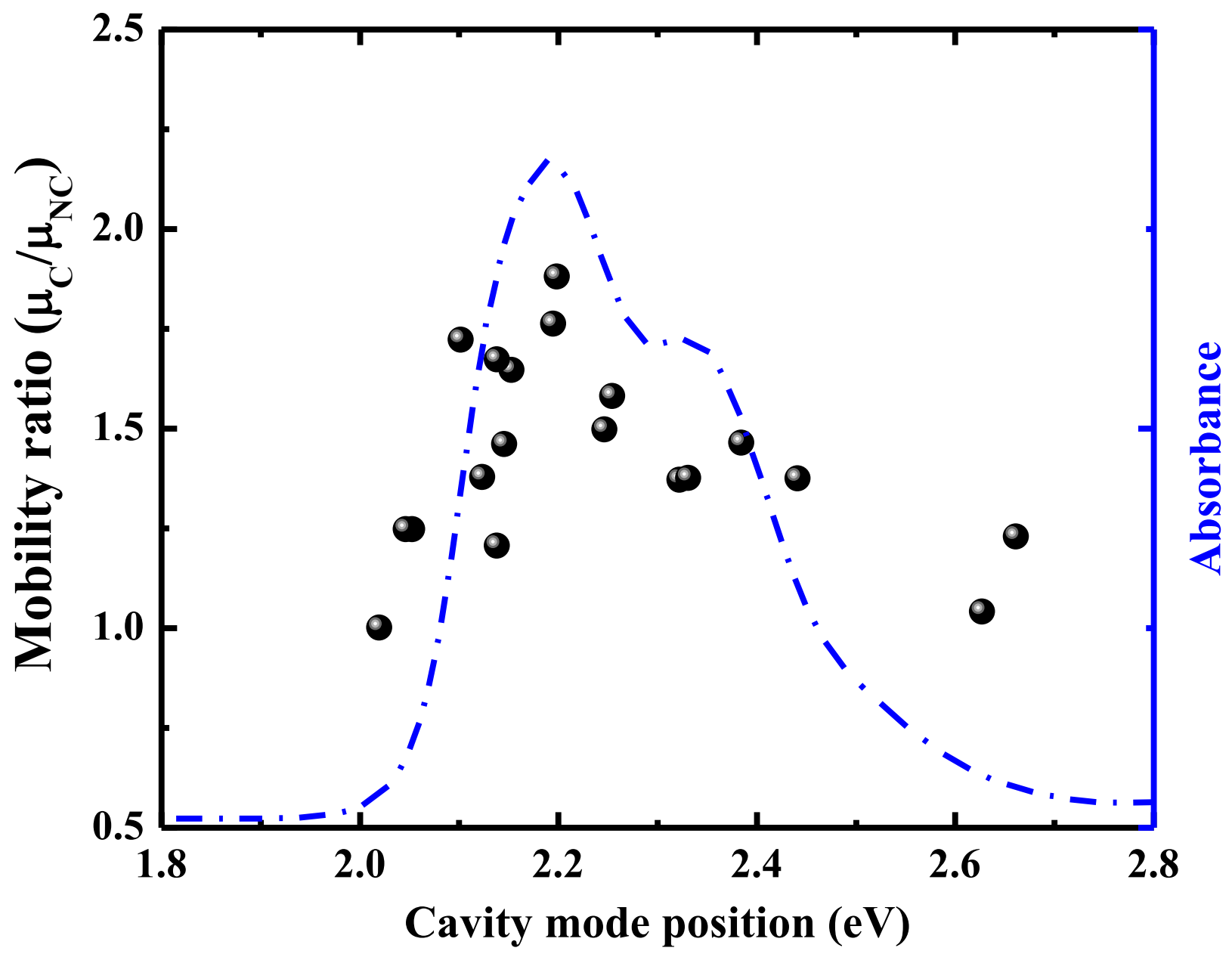

Figure S8. Variation of electron mobility ratio *versus* cavity mode position for second-order mode coupling with RhB-PDI system when PMMA spacer thickness is 40 nm.

Table S5 Cavity tuning data for the CSC experiments with RhB dye separated by a fixed PMMA spacer (40 nm).

Sr. No.	Cavity mode (nm)	μ_{NC} (10^{-3} $cm^2V^{-1}s^{-1}$)	μ_C (10^{-3} $cm^2V^{-1}s^{-1}$)	Mobility ratio (μ_C / μ_{NC})
1	466	3.466	4.261	1.229
2	472	3.735	3.892	1.041
3	508	2.402	3.305	1.375
4	520	4.389	6.429	1.465
5	532	2.676	3.683	1.376
6	534	2.466	3.380	1.371
7	550	4.955	7.832	1.581
8	552	2.024	3.031	1.498
9	564	2.629	4.946	1.881
10	565	1.909	3.366	1.763
11	576	2.466	4.060	1.646
12	578	2.640	3.856	1.460
13	580	2.242	3.753	1.674
14	580	3.370	4.064	1.206
15	584	3.266	4.502	1.378
16	590	3.283	5.654	1.722
17	604	3.222	4.021	1.248
18	606	2.528	3.155	1.248
19	614	3.468	3.473	1.001
20	696	3.508	3.511	1.001
21	712	3.183	6.019	1.891
22	734	3.256	4.136	1.270

The TMM-predicted normal-incidence spectra of the ON-resonant cavity for 230 nm PMMA are shown in Figure S9(a), with a Rabi splitting of around 0.460 eV. Strong coupling condition is achieved, and is further confirmed by the dispersion of polaritonic states (Figure S9(c)). Experimental cavity tuning spectra for some devices are shown in Figure S9(b). Cavity field

distribution profile ($|E|^2$) suggested that the third-order cavity mode is coupled as shown in Figure S9(d). The variation in electron mobility for both cavity and non-cavity structures with the cavity mode position is presented in Table S7. In another control experiment, RhB is replaced by Per-Red and found not to affect electron mobility, even when in direct contact with the active layer. The measured electron mobility ratio is presented in Table S7.

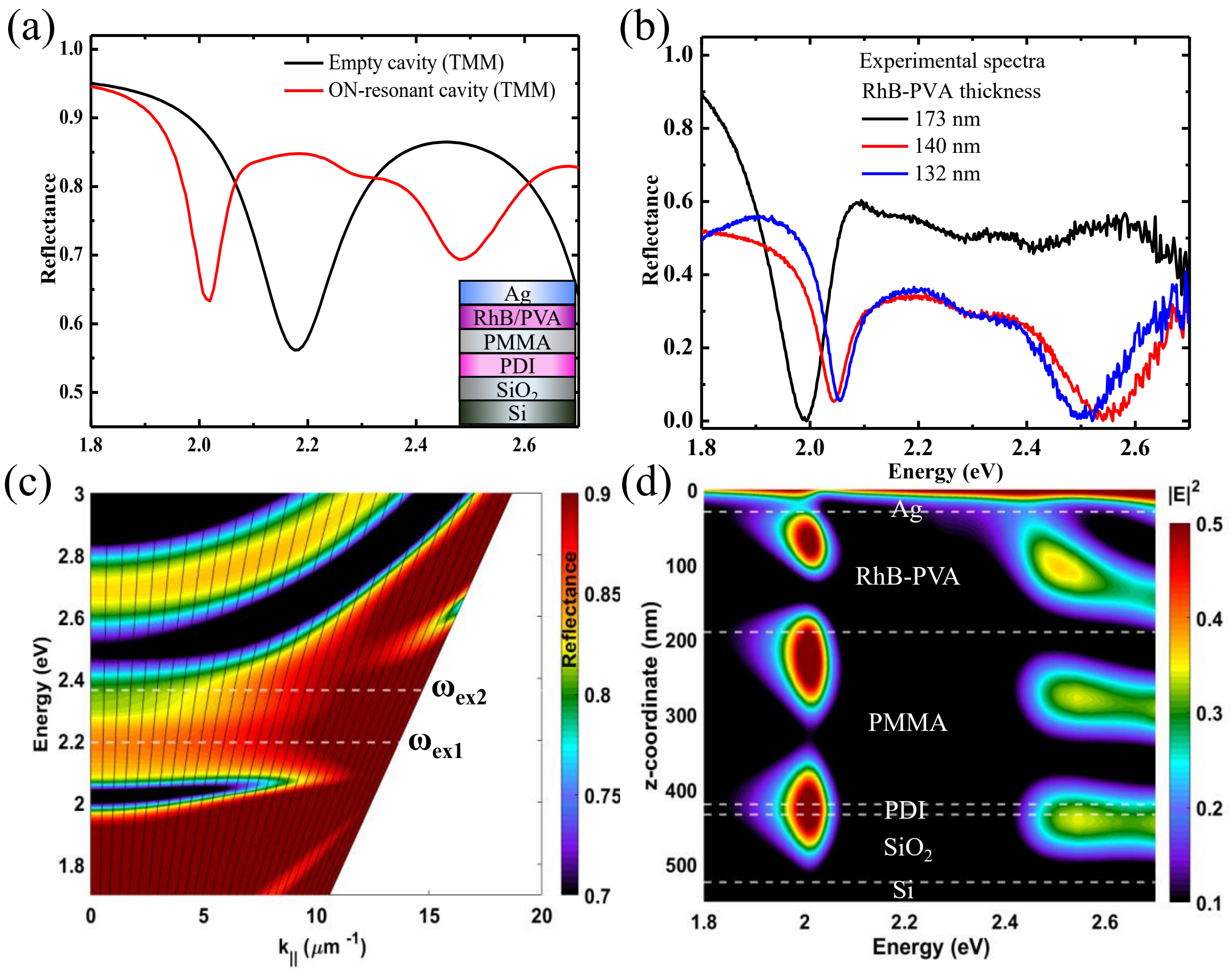

Figure S9. (a) TMM simulated spectra for the ON-resonant cavity (red) and the corresponding empty cavity spectra (black). The inset shows the multilayer structure used for the CSC experiment. (b) Experimental cavity tuning spectra obtained by varying the thicknesses of the RhB-PVA layer. (c) TMM simulated dispersion plot for the ON-resonant cavity for CSC of RhB from a third-order cavity mode. The white dashed line represents the molecular absorption band. (d) The corresponding electric field distribution ($|E|^2$) for the ON-resonant cavity within the multilayers.

Table S6: Cavity tuning data for the CSC experiments with RhB dye separated by a fixed PMMA spacer (230 nm).

Sr. No.	Cavity mode (nm)	μ_{NC} (10^{-3} cm^{2}V^{-1}s^{-1})	μ_C (10^{-3} cm^{2}V^{-1}s^{-1})	Mobility ratio (μ_C / μ_{NC})
1	490	3.161	3.368	1.065
2	502	4.240	4.639	1.094
3	504	3.019	3.548	1.175
4	536	2.541	2.712	1.067
5	545	3.478	4.145	1.192
6	586	3.003	3.265	1.086

Table S7: Cavity tuning data of CSC experiments with Perylene Red dye in direct contact with the PDI layer.

Sr. No.	Cavity mode (nm)	μ_{NC} (10^{-3} $cm^2V^{-1}s^{-1}$)	μ_C (10^{-3} $cm^2V^{-1}s^{-1}$)	Mobility ratio (μ_C / μ_{NC})
1	425	3.008	5.111	1.699
2	460	5.269	5.330	1.012
3	482	3.125	4.364	1.396
4	482	3.263	3.921	1.202
5	498	4.091	5.804	1.419
6	498	6.612	6.762	1.023
7	510	4.308	5.505	1.278
8	548	4.118	4.992	1.692
9	548	3.424	5.794	1.212
10	554	5.514	5.911	1.072
11	568	3.881	5.169	1.329
12	575	5.891	7.456	1.266
13	575	7.106	8.152	1.147
14	575	6.097	7.445	1.221
15	583	4.174	5.194	1.244
16	588	3.961	3.991	1.008
17	603	4.289	5.185	1.209
18	607	3.002	4.241	1.412
19	607	3.658	4.793	1.310
20	654	3.238	3.354	1.035

Section S4 RhB/PDI *versus* distance-dependence

RhB (diluted) in direct contact with PDI is studied to determine the nature of charge transfer occurring at the RhB-PDI interface in the absence of coupling. Thin RhB-PMMA films are prepared with a very low concentration of RhB (1% RhB and 4% PMMA in acetonitrile) to avoid the formation of leaky modes. These RhB-PMMA non-cavities are compared again with the PDI-only cavities. Sample-1 and Sample-2 are two different non-cavity devices made using 1% RhB-PMMA thin films on PDI. Their normal incidence spectra are shown in Figure S10(a), which confirms the absence of any leaky mode. The electrical characteristics for both samples are compared with their corresponding non-cavities (Figure S10 (b) and (c)). Here again, we observed some enhancement in mobility. This experiment demonstrates that even in the absence of strong coupling, interfacial charge transfer occurs at the RhB-PDI interface, thereby enhancing the electron mobility of PDI. All these measurements are done by placing PDI and RhB in direct contact.

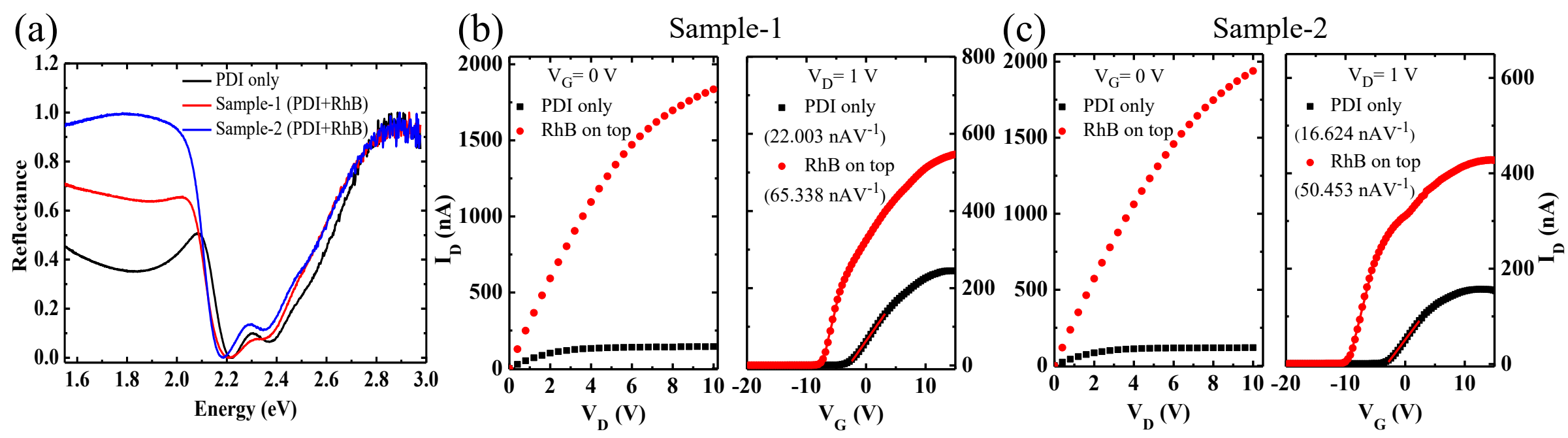

Figure S10. RhB-PMMA non-cavities without CSC. (a) Experimental normal incidence spectra for PDI only and RhB on top (sample-1 and sample-2) show negligible Rabi splitting. (b) and (c) represents the corresponding output (left panel) and transfer characteristics (right panel) for sample-1 and sample-2, respectively.

Further, Figure S11 summarises the electron mobility of PDI thin films in both non-cavity and cavity configurations as a function of PMMA spacer thickness. In the non-cavity case, incorporating RhB enhances electron mobility relative to pristine PDI in both samples, indicating improved charge transport due to interfacial interactions between RhB and PDI. Under cavity conditions, a pronounced enhancement in mobility is observed at zero spacer thickness. This effect decreases with increasing PMMA spacer thickness. This trend highlights the critical role of CSC and interfacial effects in modulating charge transport in the PDI layer.

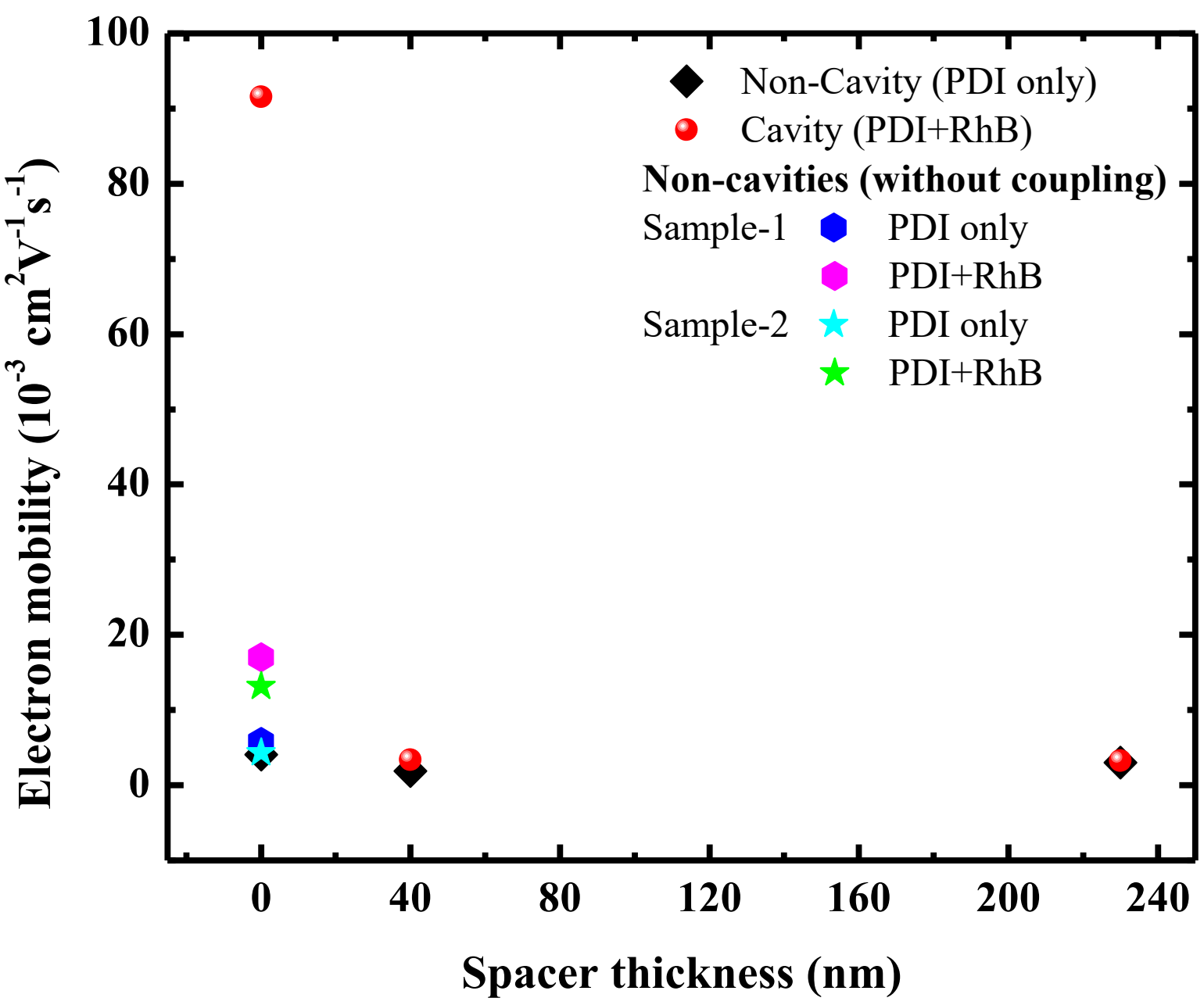

Figure S11. Electron mobility as a function of PMMA spacer thickness for cavity and non-cavity devices. The PDI-RhB cavity exhibits enhanced electron mobility compared to the corresponding non-cavity PDI, showing the effect of CSC. This effect decreases with increasing spacer thickness. Improved electron mobility in the non-cavity PDI-RhB system confirms the presence of interfacial effects.

Section S5 Hopfield coefficient, effective mass and SBH calculation

The Hopfield coefficient calculated for the lower polaritonic branch for CSC of RhB and Per-Red systems with a second-order cavity mode coupling by using the following equations [4]:

$$|\boldsymbol{C}|^2 = \frac{1}{2}\left[\mathbf{1} - \frac{(E_{ph}-E_{ex})}{\sqrt{(E_{ph}-E_{ex})^2+(\Omega_R)^2}}\right]$$

$$|\boldsymbol{X}|^2 = \frac{\mathbf{1}}{\mathbf{2}}\left[\mathbf{1} + \frac{(E_{ph}-E_{ex})}{\sqrt{(E_{ph}-E_{ex})^2+(\Omega_R)^2}}\right]$$

Here, $|\boldsymbol{C}|^2$represents photonic content, and $|\boldsymbol{X}|^2$ represents excitonic content. $\boldsymbol{E_{ph}}$ and $\boldsymbol{E_{ex}}$ are photon and exciton energies, respectively, and $\boldsymbol{\Omega_R}$ is splitting energy. $\boldsymbol{E_{ex}}$ is taken as 2.19 eV.

Hopefield coefficients calculated for the LP branch for both the cavity systems w.r.t. cavity mode tuning is shown in Figure S12 (a) and (b). Further, the relative effective mass of the lower polaritonic branch is calculated from the Hopfield coefficients by using the following relation [5]–[7]:

$$\frac{1}{m_{LP}} = \frac{|X(k_{||})|^2}{m_{ex}} + \frac{|C(k_{||})|^2}{m_c}$$

Here, m_{LP} is the effective masses of lower polaritonic states, respectively. m_{ex} and m_c are exciton mass and photon effective mass respectively. m_{ex} is considered as the vacuum electron mass only for simplicity. Photon effective mass is calculated by using following relation [5]:

$$m_c = \frac{E_c(k_{||})}{\frac{c^2}{n_c^2}}$$

Where, c is the speed of light in vacuum, and n_c is the refractive index of multilayered cavity. The relative effective mass of the lower polaritonic state with respect to the mass of the electron is calculated from optical measurements and its variation with cavity mode position is shown in Figure S12(c).

SBH is calculated using thermionic diode current equation [8],

$$I_d = AA^* e^{-e\phi_{SBH}/kT}\left(e^{eV_d/kT} - 1\right)$$

Where, I_d and V_d are drain current and drain voltage respectively, e is the electron charge, k is Boltzmann constant, T is absolute temperature, ϕ_{SBH} is SBH, and A^* is Richardson constant. From above equation SBH can be determined as follows:

$$\Phi_{SBH} = \frac{kT}{e} ln\left(\frac{AA^*\left(e^{eV_d/kT} - 1\right)}{I_d}\right)$$

Hence, difference in SBH for the cavity (Φ_C) and non-cavity (Φ_{NC}) can be calculated from above equation, at the constant gate and drain voltage as follows:

$$\Phi_C - \Phi_{NC} = \frac{kT}{e} ln\left(\frac{I_{NC}}{I_C}\frac{m_C^*}{m_{ex}^*}\right)$$

Here, $\Phi_{NC}$and Φ_C are SBH for non-cavity (PDI-only) and cavity (PDI in the presence of RhB or Per-Red), I_{NC} and I_C are the drain current at fixed biased voltage, m^*_{ex} and m^*_C are effective mass of the polaritonic state (m_{LP} for LP state) and uncoupled PDI, respectively. Please note that for simplicity m^*_{ex} is considered as the free mass of the electron.

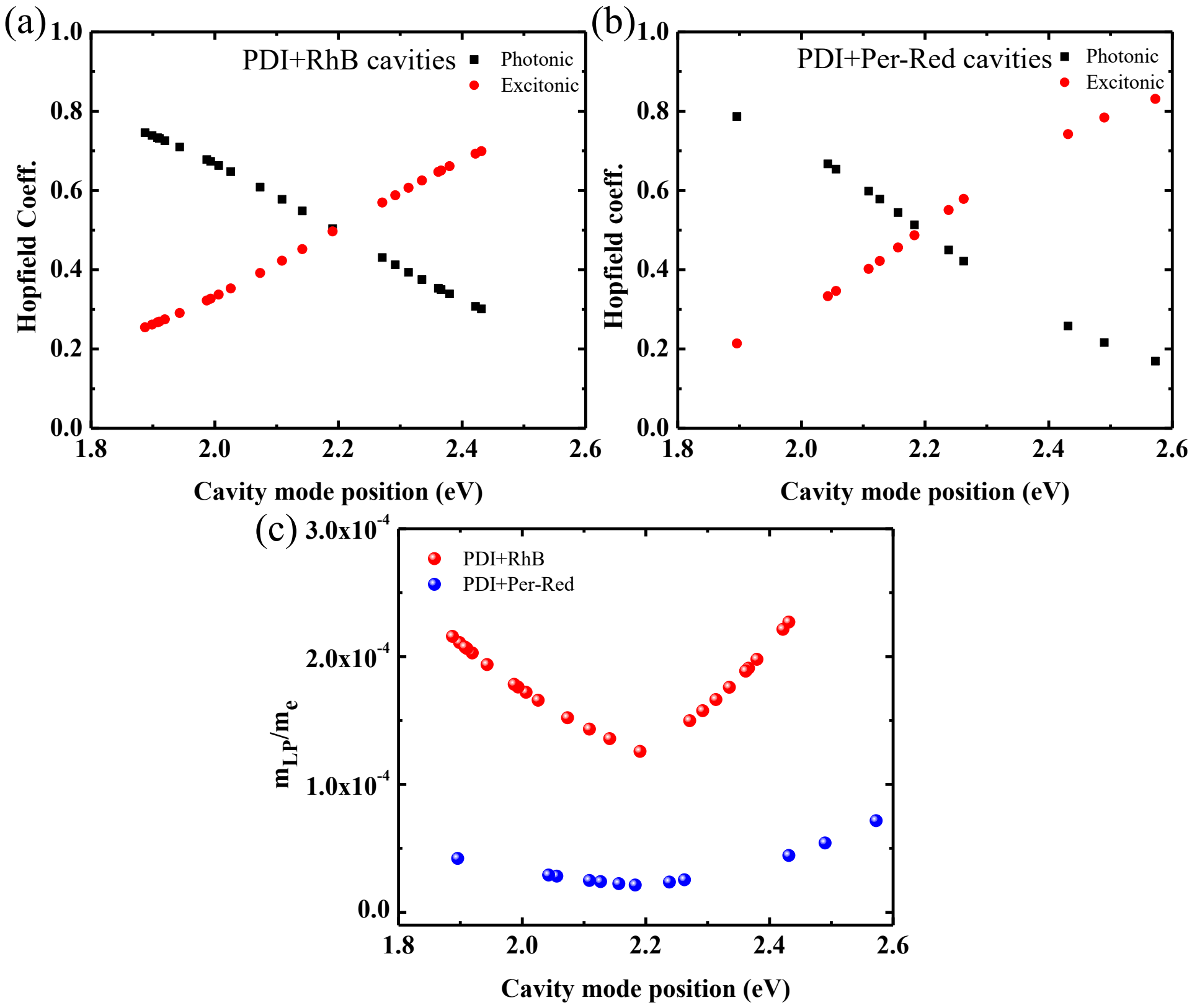

Figure S12. (a) Hopfield coefficient variation with cavity tuning for the lower polariton branch for CSC with RhB dye, and (b) with Per-Red dye. (c) Relative effective mass variation of the lower polaritonic state *versus* cavity mode position for CSC with RhB (red) and Per-Red (blue), respectively.

References

[1] K. Kaur, B. Johns, P. Bhatt, and J. George, "Controlling Electron Mobility of Strongly Coupled Organic Semiconductors in Mirrorless Cavities," *Adv. Funct. Mater.*, vol. 33, p. 2306058, 2023.

[2] R. W. Johnson, P. B. and Christy, "Optical Constants of the Noble Metals," *Phys. Rev. B*, vol. 6, pp. 4370–4379, 1972.

[3] M. N. Polyanskiy, "Refractiveindex . info database of optical constants," *Sci. Data*, vol. 11, p. 94, 2024.

[4] P. Bhatt, J. Dutta, and J. George, "Electromagnetic Field Dependence of Strong Coupling in WS2 Monolayers," *Phys. Status Solidi - Rapid Res. Lett.*, vol. 15, p. 2000580, 2021.

[5] H. Haug, "Exciton-polariton Bose-Einstein condensation," *Rev. Mod. Phys.*, vol. 82, pp. 1489–1537, 2010.

[6] Y. Sun *et al.*, "Direct measurement of polariton–polariton interaction strength," *Nat. Phys.*, vol. 13, pp. 870–875, 2017.

[7] P. Bhatt, K. Kaur, and J. George, "Enhanced Charge Transport in Two-Dimensional Materials through Light-Matter Strong Coupling," *ACS Nano*, vol. 15, pp. 13616–13622, 2021.

[8] H. Kim *et al.*, "Engineering Optical and Electronic Properties of WS 2 by Varying the Number of Layers," *ACS Nano*, vol. 9, pp. 6854–6860, 2015.
